# Glycerol Electrocatalytic Reduction Using an Activated Carbon Composite Electrode: Understanding the Reaction Mechanisms and an Optimization Study

**DOI:** 10.3389/fchem.2022.845614

**Published:** 2022-02-25

**Authors:** Siti Aqilah Nadhirah Md. Rahim, Ching Shya Lee, Mohamed Kheireddine Aroua, Wan Mohd Ashri Wan Daud, Faisal Abnisa, Patrick Cognet, Yolande Pérès

**Affiliations:** ^1^ Department of Chemical Engineering, Faculty of Engineering, University of Malaya, Kuala Lumpur, Malaysia; ^2^ Research Centre for Carbon Dioxide Capture and Utilization (CCDCU), School of Engineering and Technology, Sunway University, Bandar Sunway, Petaling Jaya, Malaysia; ^3^ Department of Engineering, Lancaster University, Lancaster, United Kingdom; ^4^ Sunway Materials Smart Science & Engineering Research Cluster (SMS2E), Sunway University, Bandar Sunway, Malaysia; ^5^ Department of Chemical and Materials Engineering, Faculty of Engineering, King Abdulaziz University, Rabigh, Saudi Arabia; ^6^ Laboratoire de Génie Chimique, Université de Toulouse, CNRS, INP, UPS, Toulouse, France

**Keywords:** indirect electrolysis, Amberlyst-15, 1,2-propanediol, reaction temperature, initial concentration, current density

## Abstract

The conversion of biomass-derived glycerol into valuable products is an alternative strategy for alleviating energy scarcity and environmental issues. The authors recently uncovered an activated carbon composite electrode with an Amberlyst-15 mediator able to generate 1,2-propanediol, diethylene glycol, and acetol *via* a glycerol electrocatalytic reduction. However, less attention to mechanistic insights makes its application to industrial processes challenging. Herein, two proposed intermediates, acetol and ethylene glycol, were employed as the feedstocks to fill the gap in the mechanistic understanding of the reactions. The results discovered the importance of acetol in producing 1,2-propanediol and concluded the glycerol electrocatalytic reduction process has a two-step reduction pathway, where glycerol was initially reduced to acetol and consecutively hydrogenated to 1,2-propanediol. At 353 K and 0.28 A/cm^2^, 1,2-propanediol selectivity achieved 77% (with 59.8 C mol% yield) after 7 h of acetol (3.0 mol/L) electrolysis. Finally, the influences of the temperature, glycerol initial concentration, and current density on the glycerol electrocatalytic reduction were evaluated. The initial step involved the C-O and C-C bonds cleavage in glycerol plays a crucial role in producing either acetol or ethylene glycol intermediate. This was controlled by the temperature, which low to moderate value is needed to maintain a selective acetol-1,2-propanediol route. Additionally, medium glycerol initial concentration reduced the hydrogen formation and indirectly improved 1,2-propanediol yield. A mild current density raised the conversion rate and minimized the growth of intermediates. At 353 K and 0.21 A/cm^2^, glycerol (3.0 mol/L) electrocatalytic reduction to 1,2-propanediol reached the maximum yield of 42.3 C mol%.

**GRAPHICAL ABSTRACT:**
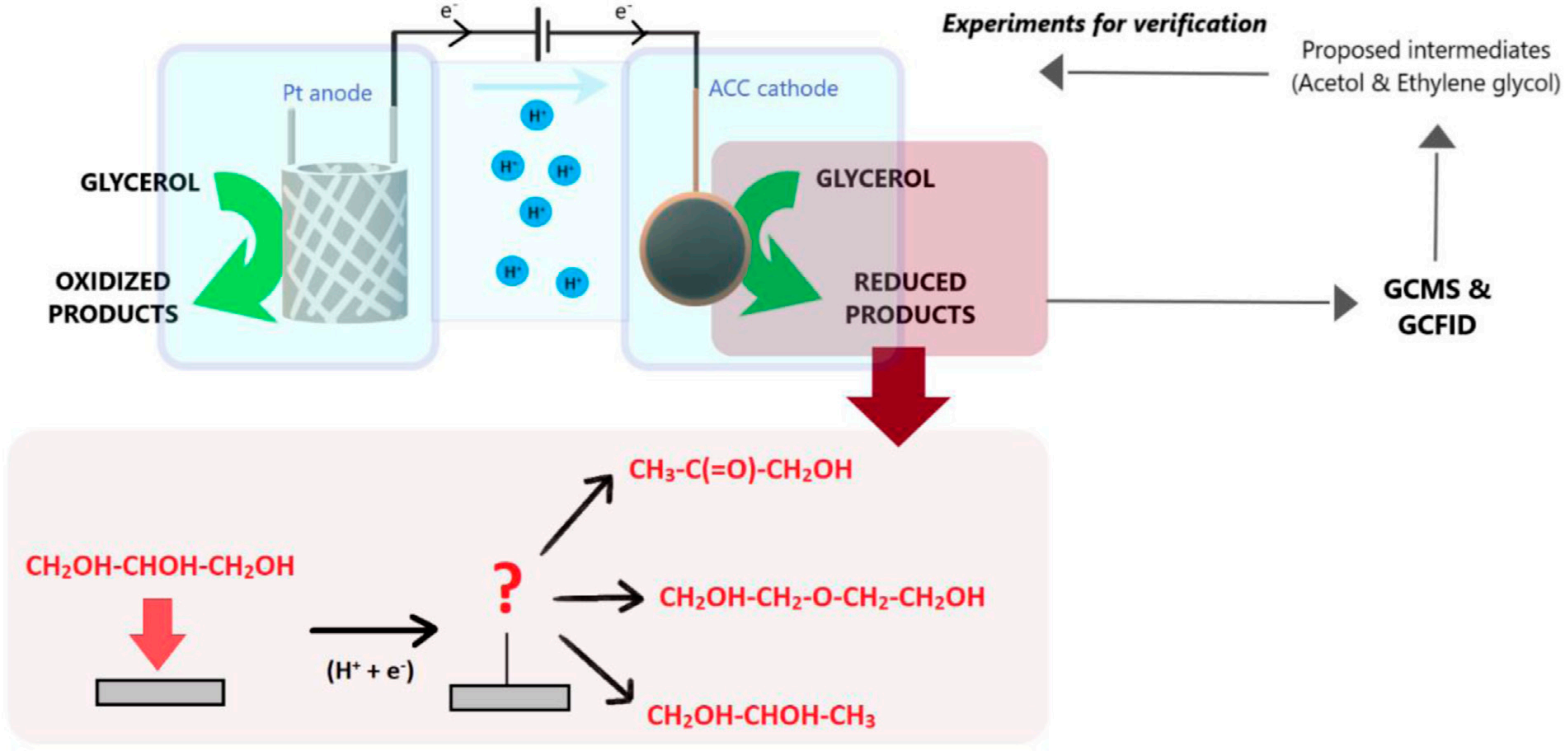


## Introduction

1,2-propanediol is an important chemical in various applications and its market growth has increased by 4% each year (Sharma et al., 2014). It is extensively employed in pharmaceutical products, food, cosmetics, unsaturated polyester resins, the preparation of paints, liquid detergents, tobacco, personal hygienic products, flavorings, and scents (Ardila et al., 2017; Dieuzeide et al., 2017). In the petrochemistry industry, 1,2-propanediol is conventionally manufactured from petroleum derivatives, specifically propylene oxide and chlorohydrin through the hydrolysis method (Dasari et al., 2005; Mitta et al., 2018; Jiménez et al., 2020). Unfortunately, the depletion of fossil fuel resources around the world has caused this route to become costly. With this in mind, there have been efforts to use biomass-derived glycerol as the feedstock in various processes, for instance, hydrogenolysis (Dieuzeide et al., 2017; Freitas et al., 2018; Cai et al., 2019; Xu et al., 2019; El Doukkali et al., 2020), and hydrodeoxygenation (Ardila et al., 2017; Yfanti and Lemonidou, 2018; Gabrysch et al., 2019). Glycerol is a waste product from biodiesel production with more than 30.8 million m^3^ produced in 2016, which was 7.5% higher than the amount produced in 2015 (Monteiro et al., 2018). Future evaluations also project biodiesel production will increase by approximately 4.5% each year and reach 41 million m^3^ in 2022, showing that more crude glycerol will be unavoidably produced. Henceforward, its usage can replace the conventional system that uses petroleum resources to generate 1,2-propanediol and simultaneously, reduces the costs of biodiesel manufacturing.

Electrochemical conversion of glycerol is yet another technique that includes general electrocatalytic oxidation and reduction reactions, which have been recently reported to outweigh the catalytic processes. Kongjao et al. (2011) were the first researchers that obtained 1,2-propanediol with other products such as ethylene glycol, acetol, propanol, 1,3-propanediol, and acrolein from glycerol electrochemical conversion. Unlike catalytic hydrogenolysis and hydrodeoxygenation practices which normally involve high temperature and high hydrogen pressure, electrochemical conversion can be done at low temperature and ambient pressure, and without the requirement of hydrogen as a reduction agent (Torres et al., 2010; Zhou et al., 2010; Sharma et al., 2014; Yfanti and Lemonidou, 2018). Instead, protons are provided by the protic electrolyte and the electrons that come from the anode part are useful as a reduction equivalent. A similar study was also continued by Hunsom and Saila research team (Hunsom and Saila, 2013, 2015; Saila and Hunsom, 2015) where so far, the highest yield for 1,2-propanediol was only approximately 15% with a Pt cathode electrode. It was proposed that glycerol was selectively oxidized in the early step, followed by the reduction reaction. The yield and selectivity were relatively minimal due to a wide distribution of products in an undivided reactor. In addition, the electrocatalytic reduction of glycerol with two primary and one secondary hydroxyl groups is particularly tricky because of their negative reduction potentials (Tanko, 2005). The reactions are complicated due to various intermediary steps and potential products. Even if 1,2-propanediol formation is achievable at low current density (0.14 A/cm^2^) in a galvanostatic mode, a selective reaction is limited by the need to untangle the reaction mechanisms (Hunsom and Saila, 2015). Each product formed through the electrochemical conversion had a different optimal electrode material, electrolyte pH, applied current density or voltage, and electrolysis time. As a result, numerous by-products were reported with 1,2-propanediol based on different electrodes and operating conditions. Without a complete understanding of the reaction mechanisms, process optimization, and possible scale-up are difficult to accomplish. Consequently, it has become an inherent impediment that must be tackled.

Primarily, the electrochemical conversion involves redox reactions in a protic environment, *e.g.*, aqueous electrolytes (Kwon et al., 2011). Therefore, it makes electrochemistry and electrochemical techniques convenient for mechanistic investigation. Voltammetric studies, specifically Tafel analysis, have been widely reported for both acidic and alkaline media; hitherto the mechanism insights were only focused on glycerol electrocatalytic oxidation, and the specific formation of intermediates and products were not determined (Habibi and Delnavaz, 2016; Ashok et al., 2018; Yahya et al., 2019). Quantum chemical computations can be employed to gain the energies of reaction intermediates and understand their interactions with the electrodes, but spectroscopic methods, *e.g.*, infrared spectroscopy, and mass spectroscopy, are also necessary to probe the mechanism in detail (Fernández et al., 2015; Garcia et al., 2016; Valter et al., 2018). Furthermore, computational techniques are unsuitable for evaluating all the pertinent factors in the experiments and thus, they cannot substitute the experimental approach for determining new electrodes and investigating the reaction mechanisms. Very recently, Sauter et al. (2017) conducted the trials by using acetol as a glycerol alternative in the electrolysis with 11 non-precious metals. The highest 1,2-propanediol selectivity (84.5%) was obtained on iron electrodes in a chloride solution with 0.09 M of acetol, resulting from acetol as the intermediate through the electrocatalytic hydrogenation.

With this motivation, the authors have introduced the reaction mechanisms of glycerol electrocatalytic reduction using an activated carbon composite (80ACC) cathode electrode in Amberlsyt-15 solution. The presence of acetol in the previous report by Lee et al. (2018) implied it was the intermediate molecule for 1,2-propanediol while diethylene glycol was speculated to be obtained from ethylene glycol intermediate. In addition to the reaction is not being explored in other articles, especially diethylene glycol formation, two proposed intermediates (acetol and ethylene glycol) were first used as the glycerol substitutes to validate the reaction mechanisms. Subsequently, the preliminary experiments on glycerol electrocatalytic reduction were performed by adjusting the reaction temperature, glycerol initial concentration, and current density. Respective to the qualitative and quantitative products distributions, both gas chromatography-mass spectrometry (GC-MS) and gas chromatography with flame ionization detection (GC-FID) were applied. This study aims to elucidate complete reaction mechanisms that govern the means of indirect glycerol electrocatalytic reduction with an activated carbon composite (80ACC) electrode. Although a few reports have revealed the reduction of glycerol to 1,2-propanediol in Amberlyst-15 solution (Nakagawa et al., 2012; Hirasawa et al., 2013; Nakagawa et al., 2014; 2018), the authors are not cognizant of any research works that have elucidated the reaction mechanisms of glycerol electrocatalytic reduction with a carbon-based electrode and Amberlyst-15 as the redox mediator. Indeed, this is also the first report on the optimization of 1,2-propanediol *via* a glycerol electrocatalytic reduction reaction with the activated carbon material electrode and Amberlyst1-5 redox mediator. The inclusive work here can be eye-opening in terms of scaling up the electrochemical technology using the inexpensive electrode and redox mediator.

## Materials and Methods

### Materials

The following chemicals were purchased and utilized without any additional purification: glycerol (99.8% purity, A. R. grade) and ethanol (greater than 95% purity, A.R. grade) were secured from R&M Chemicals, Malaysia. Amberlyst-15 hydrogen form dry, acetol (90% purity, technical grade), and polytetrafluoroethylene (PTFE) (60 wt% dispersion in H_2_O) were purchased from Sigma Aldrich, Malaysia. In addition, 1,2-propanediol (99% purity, GC grade), sodium sulfate anhydrous (Na_2_SO_4_) (greater than 99% purity, A.R. grade), tetraethylene glycol, dimethyl ether (greater than 99% purity, GC grade), and 1,3-propanediol (99% purity, GC grade) were obtained from Acros Organics, Geel, Belgium. Diethylene glycol (99% purity, GC grade) was purchased from Fluka Chemie GmbH, Buchs, Switzerland, the activated carbon (99.5% purity, an average particle size of 100 μm, and a specific surface area of 950 m^2^/g) was purchased from Sigma Aldrich, St. Louis, MO, and the carbon black (99% purity, an average particle size of 13 nm, and a specific surface area of 550 m^2^/g) was purchased from Alpha-Chemicals Sdn Bhd, Penang, Malaysia.

### Cathode Electrode Synthesis

The activated carbon composite (80ACC) (with a geometrical surface area of 7.1 cm^2^) was synthesized as the cathode electrode using the earlier technique (Lee et al., 2018). 80% (weight) activated carbon and 20% (weight) carbon black of 1.0 g total weight were mixed. A binder solution of 80% (v/v) 1,3-propanediol and 20% (v/v) polytetrafluoroethylene (PTFE) was further blended with the pre-mixed powder using a 2:1 ratio by mortar and pestle for 25 min 373 K (2 h), 453 K (1 h), 523 K (1 h), and 623 K (30 min) drying order was applied on the resulting paste in a furnace, which was respective to the heating rates of 0.8, 1.3, 1.2, and 3.3 K/min. When the sintering process is completed, the copper wire was attached to the electrode as a current collector and covered by the organic adhesive.

### Reaction Mechanisms’ Validation Using the Proposed Intermediates

Two proposed intermediates, *e. g.*, acetol and ethylene glycol, were assessed as the starting materials to verify the suggested reaction mechanisms. As the 80ACC electrode is feasible to obtain 1,2-propanediol, 80ACC was employed as cathode and Pt mesh cylinder (with 22 cm^2^ of geometrical surface area) as anode electrodes for acetol electrolysis in a two-compartment reactor. The variation of kinetics parameters was carried out to evaluate their effects on the products distribution. In the first stage, to prove 1,2-propanediol can be attained from acetol, each compartment contained 0.25 L of 0.3 mol/L acetol and 9.6% (w/v) of Amberlyst-15 in 0.3 mol/L of sodium sulfate (Na_2_SO_4_) (pH 1). At 350 rpm of constant stirring speed and 0.14 A/cm^2^ of current density, the reaction temperature was altered from 300 to 326.5, 353, and 379.5 K under the air atmosphere. The experiments were carried out for 8 h and the sample was manually taken every 1 h. To investigate the influence of initial concentration, the concentrations of 0.3, 1.65, 3.0, and 4.35 mol/L were implied. 0.14, 0.21, 0.28, and 0.35 A/cm^2^ of current densities (respective to 1.0, 1.5, 2.0, and 2.5 A current) were used to evaluate its effect on the production of 1,2-propanediol. Next, to validate ethylene glycol produce diethylene glycol, 0.1 L of ethylene glycol was loaded in a one-compartment reactor, without the application of electrical current. The optimum condition from acetol experiments was applied for this verification. The investigation was conducted for 8 h. Lastly, at the same condition, glycerol was used as the starting material for electrolysis without electricity to demonstrate acetol production.

### Preliminary Experiments: Electrocatalytic Reduction of Glycerol

The same two-compartment reactor, anode, and cathode electrodes were utilized for the glycerol electrocatalytic reduction experiments. Both parts were filled with 0.25 L of 0.3 mol/L pure glycerol. An acidic solution of 9.6% (w/v) of Amberlyst-15 in 0.3 mol/L of Na_2_SO_4_ (pH 1) was used as the electrolyte. The work was divided into a few parts to study the effects of each kinetics parameter. In the first part, with 0.3 mol/L of glycerol as the feedstock, the temperature was regulated from room temperature (300 K) to 326.5, 353, and 379.5 K under the air atmosphere. The current density was 0.14 A/cm^2^ at 350 rpm constant stirring rate. In every 1 h, 5 ml of sample was manually acquired and prepared for the characterization using the gas chromatography with mass spectroscopy instrument (GC-MS). The quantification of the presented compounds was done with gas chromatography–flame ionization detector (GC-FID). The next batch of experiments involved the variation of glycerol initial concentration (0.3, 1.65, 3.0, and 4.35 mol/L). With the optimal reaction temperature and initial concentration of glycerol, the current density was varied (0.07, 0.14, 0.21, and 0.28 A/cm^2^); applied current of 0.05, 1.0, 1.5, and 2.0 A. All the experiments were conducted in batch mode.

### Analytical Techniques

A gas chromatography (Agilent Model 7890, United States) with mass spectroscopy (GC-MS) was utilized for the characterization of liquid products. The DB-Wax capillary column with 30 m length and 0.25 mm inner diameter with 0.25 µm film thickness (Phenomenex, United States) was employed. The oven temperature was programmed to start at 318 K for 5 min and ramped to 513 K at 10 K min^−1^ with a final hold time of 5 min. To prepare the sample, 1000 µl of the liquid sample was mixed with 1000 µl of internal standard (tetraethylene glycol) and ethanol was added to make up to 10 ml of solution. The obtained sample was neutralized using sodium hydroxide and filtered with a 0.45 µm nylon syringe. 1 µl of prepared sample was injected in the GCMS and helium (>99.99% purity) with a constant flow rate of 2.0 ml min^−1^ was used as a carrier gas. The acquired peaks of compounds were compared with the MS library (Agilent, ChemStation software) and chemical standards. For quantification of the presented compounds, a gas chromatography (GC) (Model 6890, Agilent) connected to a flame ionization detector (FID) and attached with the same capillary column was employed. The analysis was conducted under identical conditions as GC-MS analysis. The integrated peak areas calculation was made based on the standards calibration curves plotted with known concentration. The conversion of glycerol, products yield, and selectivity were then calculated using Eqs 1–3, respectively.

$$\text{Glycerol conversion}\left(\text{\%}\right)=\frac{\text{Converted glycerol}\left[\text{Gly}.\text{ in feed}-\text{Gly}.\text{ in outlet }\left(\text{in C mole}\right)\right]}{\text{Total amount of glycerol in feed}\left(\text{in C mole}\right)}\times 100\text{\%}$$ (1)

$$\text{Product yield}\left(\text{\%}\right)=\frac{\text{Amount of product}\left(\text{in C mole}\right)}{\text{Total amount of glycerol in feed}\left(\text{in C mole}\right)}\times 100\text{\%}$$ (2)

$$\text{Product selectivity}\left(\text{\%}\right)=\frac{\text{Amount of product}\left(\text{in C mole}\right)}{\text{Converted glycerol}\left[\text{Gly}.\text{ in feed}-\text{Gly}.\text{ in outlet}\left(\text{in C mole}\right)\right]}\times 100\text{\%}$$ (3)

*Glycerol can be replaced with the intermediates: acetol and ethylene glycol.

## Results and Discussion

### Reaction Mechanisms Elucidation: Acetol as the Reactant in the Electrolysis

**FIGURE 1:**
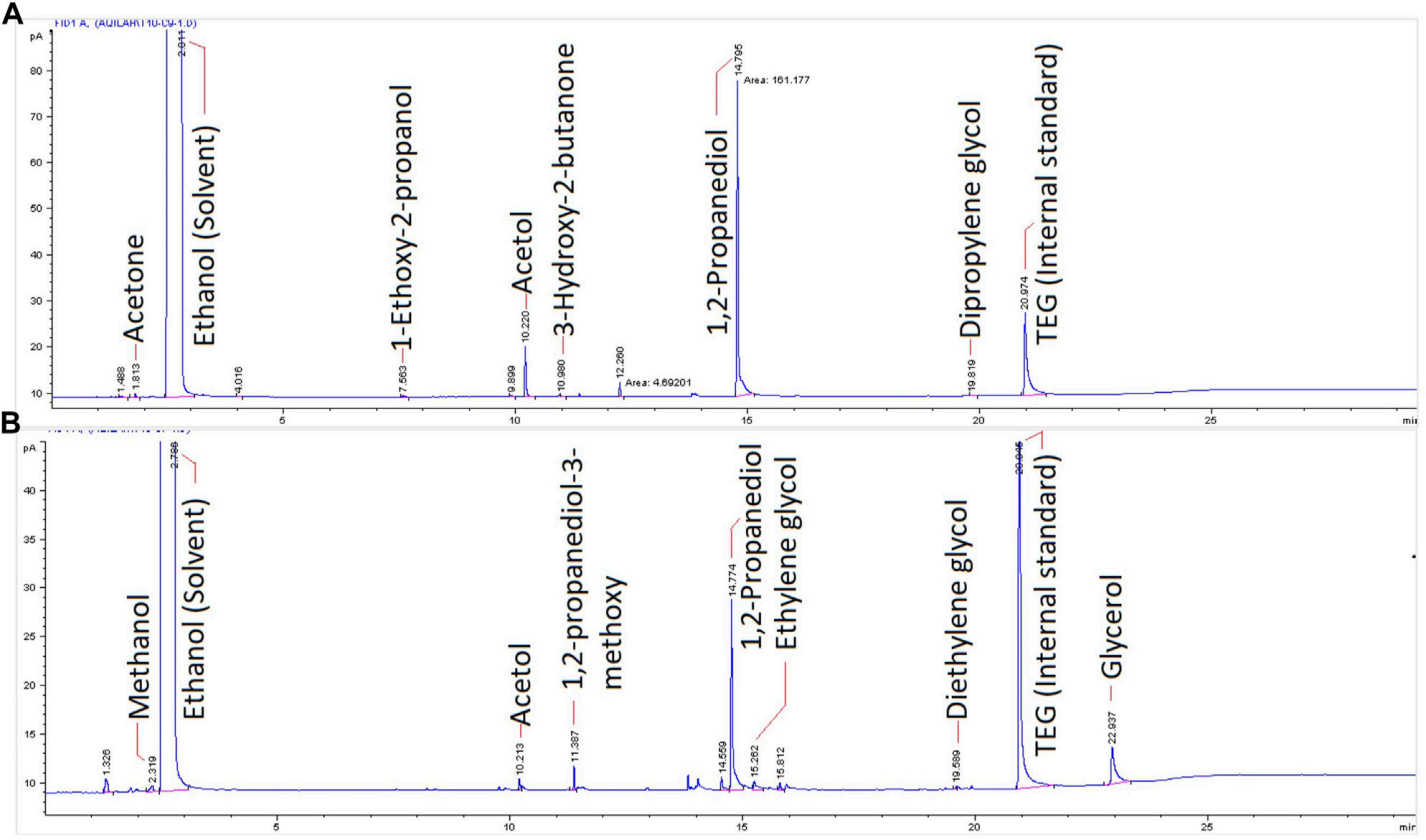
GC chromatograms after 8 h of **(A)** acetol electrolysis and **(B)** glycerol electrocatalytic reduction.

**SCHEME 1:**
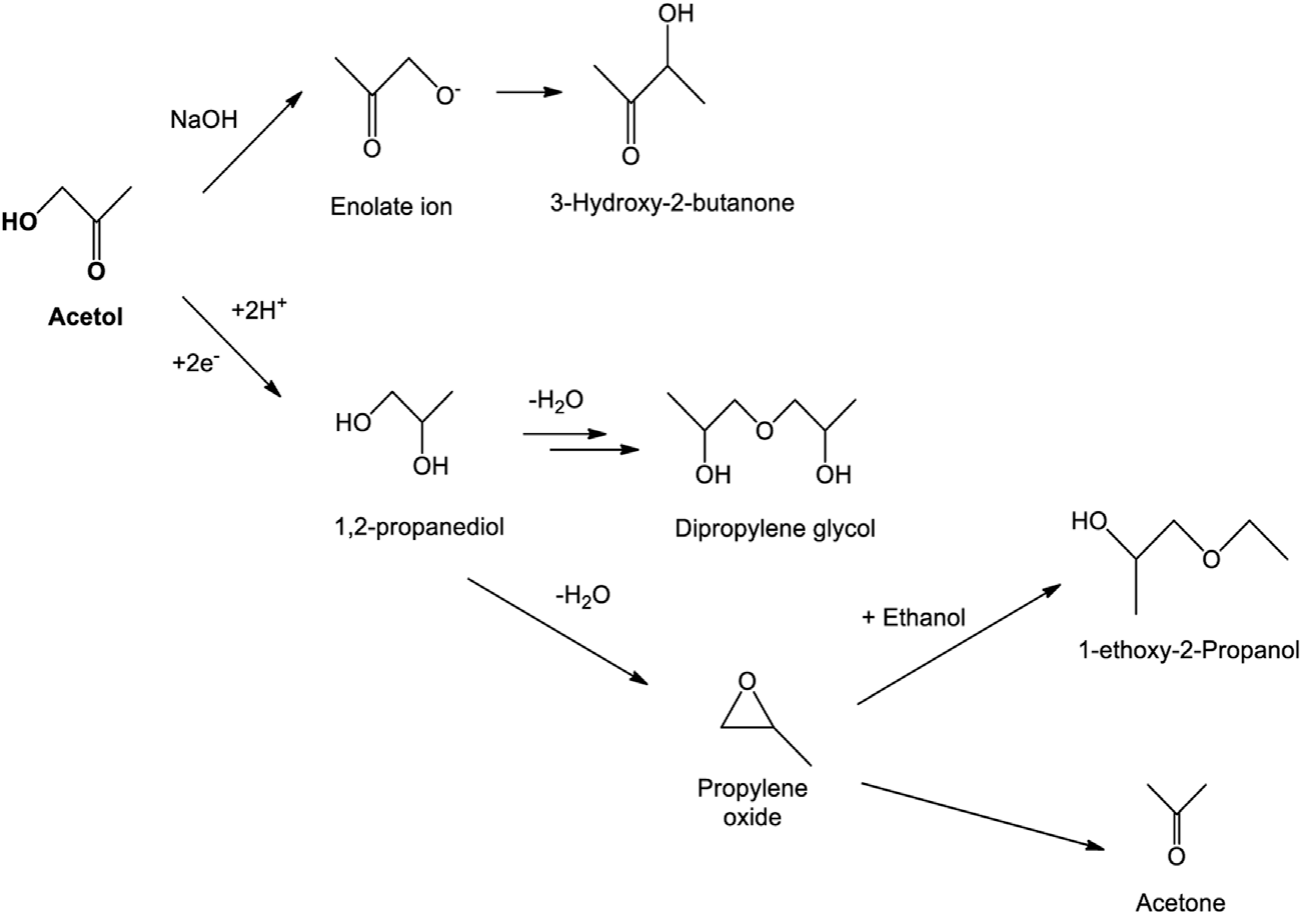
Formation of 1,2-propanediol and by-products from acetol electrolysis.

When acetol was utilized as the reactant, Figure 1A shows 1,2-propanediol as a major product with other by-products including dipropylene glycol, 1-ethoxy-2-propanol, and 3-hydroxy-2-butanone. Acetone was detected only in traces below 353 K of temperature due to its fast volatility at a high temperature (Sauter et al., 2017). The suggested reaction mechanisms for 1,2-propanediol and by-products formation are presented in Scheme 1. 1,2-propanediol was attained through the electrocatalytic hydrogenation pathway. In other words, acetol was proved to be the intermediary compound for 1,2-propanediol. Intermolecular and intramolecular dehydration of 1,2-propanediol occurred in an acidic medium and developed dipropylene glycol and propylene oxide, correspondingly. Propylene oxide isomerization led to other minor products formation, namely, acetone and 1-ethoxy-2-propanol (Yu et al., 2009). In the presence of the basic compound, *e.g.*, ethanol, the propylene oxide ring favorably opened at the C-O bond with a less sterically hindered position and dominated secondary alcohol (1-ethoxy-2-propanol) formation (Zhang et al., 2005; Zhang et al., 2014). Zhang et al. (2016) successfully produced 1-ethoxy-2-propanol by alcoholysis of propylene oxide and ethanol in the presence of catalyst whilst Chitwood and Freure (1946) acquired it even without any catalyst. In great support from the later work, 1-ethoxy-2-propanol was indirectly generated through this reaction during the sample preparation and GC-MS characterization that incriminated ethanol as the solvent (Chitwood and Freure, 1946; Ślipko and Chlebicki, 1981). During the sample preparation, sodium hydroxide (NaOH) was used to precipitate out the sodium sulfate electrolyte from the taken sample. With this fact, under alkaline conditions, carbonyl compound like acetol with α-CH (H-Cα-C=O) bond is a reactive compound, hence, it reacted with this base to form an enolate ion. Swiftly, it produced 3-hydroxy-2-butanone as a side product (Heathcock, 2014). Its formation was not only as a minor product but also from the sample neutralization *via* the intermolecular aldol-condensation mechanism.

#### Kinetics Model and Effects of Kinetics Parameters

**FIGURE 2:**
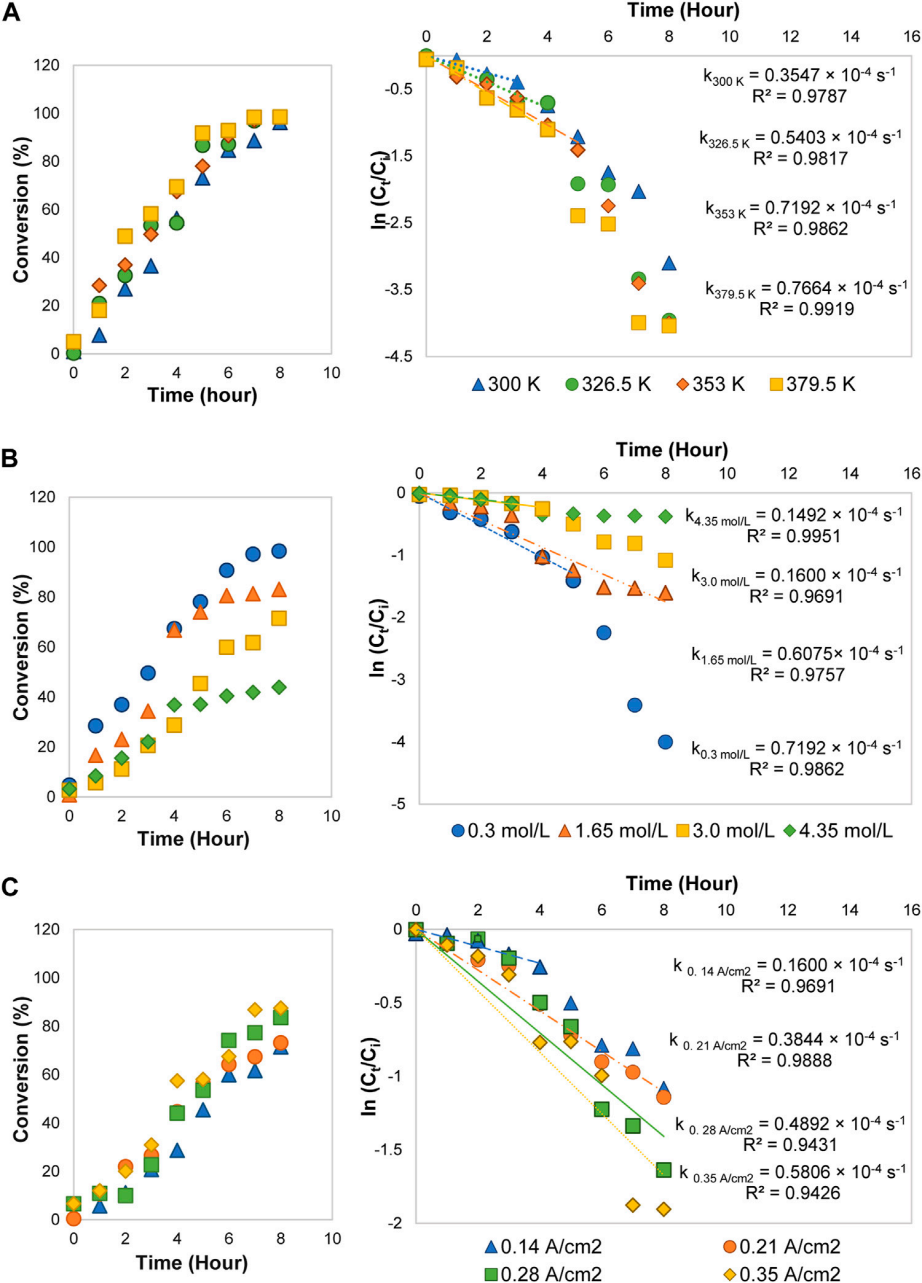
Conversions and first-order kinetics models of acetol electrocatalytic reduction at different **(A)** reaction temperatures, **(B)** initial concentrations, and **(C)** current densities.

The kinetics parameters *e. g.*, reaction temperature, initial concentration, and current density were later varied to examine their effects on 1,2-propanediol formation. As shown in Table 1, 1,2-propanediol was a dominant product, although some conditions were affected by the high production of the by-products. All the conditions were fitted to the first-order kinetics model during the first 3–8 h of electrolysis time. The plots obtained from ln (C_t_/C_o_) *vs* time graphs were in linear form (Figure 2). Its integral expressions are displayed in Eqs 4, 5, where *C*_*t*_ is the instantaneous concentration of glycerol, *C*_*o*_ is glycerol initial concentration, *t* is time, and the slope provides *k* is the kinetics rate constant of the reactions. The consistency between experimental data and the model-predicted values was expressed by the determination coefficients (*R* ^*2*^, values closeness to 1) with *R* ^*2*^ larger than 0.9426. The graphs of acetol conversion are also given in Figure 2.

$$C_{t}=C_{o}\;e^{-kt}$$ (4)

$$\ln C_{t}=\ln C_{o}-kt$$ (5)

**TABLE 1:** 1,2-propanediol and minor products selectivity and yield under different conditions after 8 h of reactions (anode: Pt, electrolyte: 0.3 mol/L Na_2_SO_4_ + 9.6% (w/v) Amberlyst-15).

[Acetol] (mol/L)	*j* (A/cm^2^)	E (V)	T (K)	Acetol conversion		1,2-Propanediol		Dipropylene glycol		1-ethoxy-2-propanol		Acetone		3-hydroxy-2-butanone	
				(%)	k (s^−1^)	Y	S	Y	S	Y	S	Y	S	Y	S
						(%, in C mol)									
Effect of reaction temperature															
0.30	0.14	18.2	300.0	96	0.3547 × 10^–4^	9.2	10	—	—	0.6	0.6	—	—	0.04	0.4
0.30	0.14	15.9	326.5	98	0.5403 × 10^–4^	15.8	16	5.0	5	2.8	3	1.4	1.4	0.08	0.08
0.30	0.14	13.1	353.0	98	0.7192 × 10^–4^	28.9	29	10.9	11	9.1	9	—	—	3.8	4
0.30	0.14	11.7	379.5	99	0.7664 × 10^–4^	19.6	20	12.8	13	7.6	8	—	—	3.6	4
Effect of acetol initial concentration															
0.30	0.14	15.8	353.0	98	0.7192 × 10^–4^	28.9	29	11.0	11	9.0	9	—	—	3.8	4
1.65	0.14	19.8	353.0	83	0.6075 × 10^–4^	37.8	46	11.0	13	5.0	6	—	—	3.5	4
3.00	0.14	20.3	353.0	72	0.1600 × 10^–4^	42.5	59	4.1	6	4.0	6	—	—	2.8	4
4.35	0.14	21.1	353.0	43	0.1492 × 10^–4^	20.2	46	4.8	11	4.0	9	—	—	2.3	5
Effect of current density															
3.00	0.14	21.0	353.0	72	0.1600 × 10^–4^	42.5	59	4.1	6	4.0	6	—	—	2.8	4
3.00	0.21	22.4	353.0	73	0.3844 × 10^–4^	44.7	61	7.4	13	3.7	5	—	—	3.7	5
3.00	0.28	25.7	353.0	84	0.4892 × 10^–4^	47.3	57	14.0	17	4.9	5	—	—	2.4	3
3.00	0.35	30.4	353.0	87	0.5804 × 10^–4^	37.7	43	15.0	18	13.1	16	—	—	2.5	3

[Acetol], Acetol concentration; *j*, Applied current density; E, Applied voltage; T, Reaction temperature; k, Kinetics rate constant; Y, Yield; S, Selectivity.

#### Effect of Reaction Temperature

At 0.3 mol/L of acetol concentration, when the temperature was modified in the range of 300–379.5 K, the acetol conversion was enhanced up to 99% with a kinetics rate constant of 0.7664 × 10^–4^ s^−1^ (379.5 K) (Figure 2A). Although 1,2-propanediol demonstrated a higher yield and selectivity than other products for all temperatures, however, the yield of total products from the converted glycerol could not accomplish 100% in C mol. It shows that hydrogen evolution reaction (HER) is the prominent reaction at low acetol concentration regardless of the temperature used in the system. The formed hydrogen may carry the compounds with high volatility like acetone and acetol out from the reactor. Literature surmised that low pH also slightly contributed to a dictating impact of HER (Sauter et al., 2017). H^+^ ions from the anode part compete with acetol for the redox reaction and were reduced into hydrogen instead of only 1,2-propanediol. 1,2-propanediol yield increased with the temperature improvement and achieved the highest value (28.9 C mol%) at 353 K. Besides, the by-products yield was rapidly boosted and sparked the reduction in 1,2-propanediol selectivity at the higher temperature, showing its significant role in controlling the formation of by-products.

#### Effect of Acetol Initial Concentration

1,2-propanediol formation was preferred at 353 K, hence, this temperature was used for the effect of acetol initial concentration study. High initial concentration commonly allows to proscribe the competing HER and liquid products generation in the electrolysis, consequently, it can encourage 1,2-propanediol production (dos Santos et al., 2015). The electrolysis was then carried out with 0.3, 1.65, 3.0, and 4.35 mol/L concentrations. As tabulated in Table 1, the 1,2-propanediol yield was increased up to 42.5 C mol% (59% selectivity) when the concentration of acetol was adjusted from 0.3 to 3.0 mol/L. The increasing pattern illustrated the importance of acetol as an intermediate to form 1,2-propanediol. The availability of acetol molecules to react with protons and electrons improved 1,2-propanediol yield at a higher concentration. Beyond 3.0 mol/L, it was accompanied by side effects, where 1,2-propanediol and minor products yields were dropped. It was due to the high viscosity of the solution that led to the poisoning of the 80ACC cathode electrode surface (Nascimento and Linares Leon, 2014). The conversion rate was also decreased in Figure 2B, influenced by the reduction of conductivity in the electrolyte solution. A decline of protons in the aqueous electrolyte prevented the main acetol reaction (electrocatalytic hydrogenation to 1,2-propanediol). The rate was reduced from 0.7192 × 10^–4^ s^−1^ (0.3 mol/L) to 0.1492 × 10^–4^ s^−1^ with too high acetol concentration (4.35 mol/L). Moreover, a higher applied potential will be needed to maintain the current density and number of electrons for acetol electrocatalytic hydrogenation, resulting in low-performance efficiency. Therefore, 3.0 mol/L is the most suitable acetol concentration for the electrolysis.

#### Effect of Current Density

In an electrochemical process, the reaction rate is another important factor that is directly determined by the current density parameter. At the optimum temperature (353 K) and acetol initial concentration (3.0 mol/L), four different electric currents, namely, 1.0, 1.5, 2.0, and 2.5 A correspond to 0.14, 0.21, 0.28, and 0.35 A/cm^2^ current densities were applied. 0.14 A/cm^2^ was chosen as the minimum value because 1,2-propanediol was only detected at this current density and above from the previous studies (Hunsom and Saila, 2013; 2015).In agreement with Faraday’s law, the conversion rate was improved from 0.1600 × 10^–4^ s^−1^ (0.14 A/cm^2^) to 0.5806 × 10^–4^ s^−1^ at 0.35 A/cm^2^ (Figure 2C). Here, the yield of 1,2-propanediol was gradually boosted until 47.3 C mol% (eighth hour) at 0.28 A/cm^2^ and vaguely decreased at 0.35 A/cm^2^. Higher the electricity input accelerated hydrogen ions and electrons transportation rates, resulting in a notable 1,2-propanediol selectivity and yield. In contrast, additional growth in current density to 0.35 A/cm^2^ diminished 1,2-propanediol yield and selectivity. In fact, high external energy from this electricity allowed the decomposition of 1,2-propanediol into other minor products. 0.35 A/cm^2^ developed the highest yield values of 1-ethoxy-2-propanol (13.1 C mol%) and dipropylene glycol (15.0 C mol%). Furthermore, these by-products were also formed in large amounts at longer reaction time as presented in Supplementary Figure S1. The maximum yield (59.8 C mol%) and selectivity (77%) for 1,2-propanediol reached at the seventh hour (at 0.28 A/cm^2^) and decreased at the eighth hour because 1,2-propanediol was converted into 1-ethoxy-2-propanol (4.9 C mol% yield) and dipropylene glycol (14.0 C mol% yield).

To sum up the above discussions, acetol is an essential compound to generate 1,2-propanediol through the electrocatalytic hydrogenation on the 80ACC cathode electrode. On a similar note, there is no ethylene glycol or diethylene glycol present, confirming diethylene glycol was not from acetol intermediate. This point is affirmed during ethylene glycol dehydration in a one-compartment reactor in the next discussion (Section 3.2).

### Ethylene Glycol and Glycerol Reactions Without the Electrical Current

**FIGURE 3:**
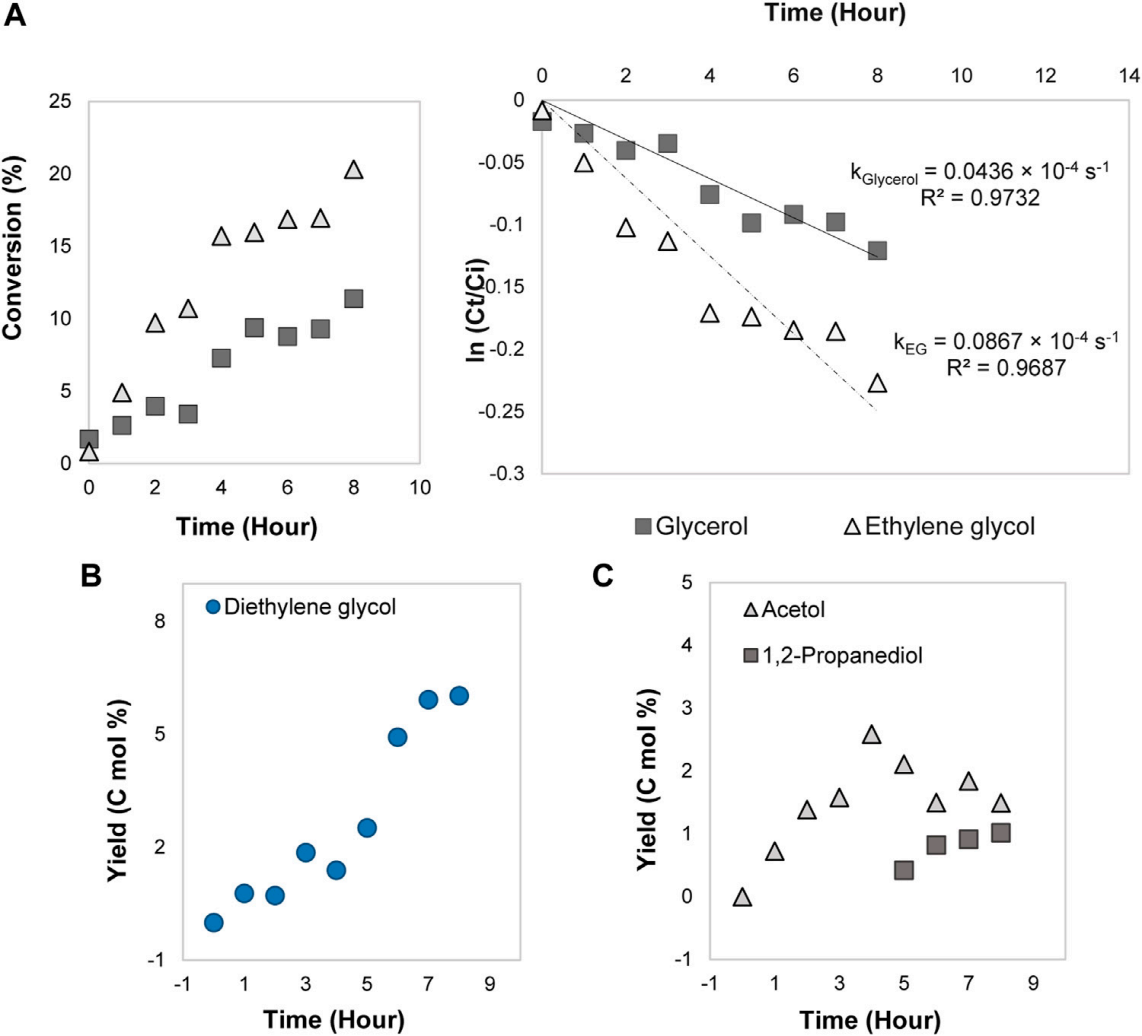
**(A)** Conversions and first-order kinetics models for ethylene glycol and glycerol reactions. Products distributions from **(B)** ethylene glycol and **(C)** glycerol dehydration.

The reactions of ethylene glycol and glycerol under the optimum temperature (353 K) and initial concentration (3.0 mol/L) were conducted in the absence of electricity. These experiments were carried out to prove that ethylene glycol can produce diethylene glycol while glycerol generates acetol through the dehydration mechanism route. By employing ethylene glycol and glycerol in the absence of electricity, their conversions were detected at 353 K with minimal conversion rates. From Figure 3A, the conversion for glycerol (11%) with a kinetics rate constant of 0.0436 × 10^–4^ s^−1^ was lower than ethylene glycol (20%, 0.0867 × 10^–4^ s^−1^) at 353 K. Chimentão et al. (2021) exhibited a small glycerol conversion (30%) in dehydration system at low temperature (463 K) while other reports (Cecilia et al., 2015; Dalla Costa et al., 2016; Ma et al., 2016; Célerier et al., 2018) achieved high conversion (above 85%) at high temperature (above 573 K). It is, therefore, conceivable to conclude that dehydration of glycerol is not only required a good catalyst but also needs high external energy in the experimental conditions to initiate and speed up the reaction. The poorer glycerol conversion than ethylene glycol attributed to more hydrogen bond in its molecule has higher activation energy barrier for the transformation to the value-added compounds.

Figure 3B illustrates diethylene glycol yield was enhanced with the increase of reaction time and the maximum value was 6.0 C mol%. It proves dehydration reaction occurred with the aid of Amberlyst-15 (rich in Brønsted acid sites) under high temperature similar to Lei et al. (2021) work, where diethylene glycol was observed as a by-product on Co/γ-Al_2_O_3_ catalyst that consisted high Brønsted acid sites. In Figure 3C, acetol appeared as the only product until the fourth hour with the maximum yield of 2.6 C mol%. This outcome surpassed Kongjao et al. (2011) work, where they obtained acetol, acrolein, and 2-propene as products in sulfuric acid electrolyte. With Amberlyst-15 as an acidic medium, the glycerol dehydration became more selective to acetol, and the reaction mechanism is more promising. This is because acetol is prevalent in this pathway and is an important intermediate in achieving high 1,2-propanediol selectivity. Other researchers also showed Amberlyst co-catalyst enabled the generation of 1,2-propanediol through acetol intermediate which corroborates our results (Miyazawa et al., 2006; Miyazawa et al., 2007a; b). In this report, the acetol yield was relatively low and slightly dwindled at the fifth hour and 1,2-propanediol started to produce even without the presence of hydrogen. Since the only source of reduction agent to form 1,2-propanediol was from another acetol or glycerol molecule, Chiu et al. (2006) assumed the scavenging of hydrogen from glycerol happened and it was used as a source to produce 1,2-propanediol. It agrees with our present study, though 1,2-propanediol yield (around 1.0 C mol%) was insignificant. Besides, the absence of ethylene glycol and diethylene glycol in this reaction reinforced the formation of ethylene glycol as the intermediate involved electrolysis mechanism on the 80ACC electrode. Overall, the mechanistic investigation here validated that acetol produced 1,2-propanediol through the electrocatalytic hydrogenation, whereas ethylene glycol and glycerol generated diethylene glycol and acetol, respectively *via* dehydration reaction.

### Identified Reaction Mechanisms

Generally, the direct electrochemical conversion of aqueous glycerol includes oxidation and reduction reactions at the anode and cathode electrodes, correspondingly. From the literature, glycerol electrocatalytic oxidation and oxygen evolution reaction (OER) simultaneously happened at the anode (1–5) instigating more than one intermediate adsorbed on the electrode surface (Simões et al., 2012; Pagliaro, 2017; Talebian-Kiakalaieh et al., 2018). The adsorbed glycerol species (M-C_3_H_8_O_3ads_) on the electrode surface (M) interact with an adsorbed hydroxyl group (M-•OH_ads_) to oxidize glycerol into intermediates/products and CO_2_ through Langmuir-Hinslewood mechanism (4) (Gonçalves et al., 1985; Gomes et al., 2013). The intermediates/products produced depend on the essence of the electrodes and operating conditions used in the electrolysis system (Rahim et al., 2020). At the cathode, it is acknowledged in most studies that only protons from the anodic compartment are reduced into hydrogens (6) without considering glycerol electrocatalytic reduction reaction (GERR). The total reactions for a complete glycerol electrochemical conversion can be written as (7).

Anode:

M + C_3_H_8_O_3_ → M-C_3_H_8_O_3ads_ → Intermediates/products + H^+^ + e^−^_*(Partial electrocatalytic oxidation)*_ (1)
M + C_3_H_8_O_3_ → 3 M-CO_ads_ + 8H^+^ + 8e^−^_*(Complete glycerol electrocatalytic oxidation)*_ (2)
M + H_2_O → M-OH_ads_ + H^+^_*(Formation of hydroxyl group via oxygen evolution reaction)*_ (3)
CO_ads_ + •OH_ads_ → CO_2_ + H^+^ + e^−^_*(Langmuir-Hinshelwood mechanism)*_ (4)
C_3_H_8_O_3_ + 3H_2_O → 3CO_2_ + 14H^+^ + 14e^−^_*(Complete glycerol electrocatalytic oxidation*)_ (5)

Cathode:

14H^+^ + 14e^−^ → 7H_2__*(Hydrogen evolution reaction)*_ (6)

Overall:

C_3_H_8_O_3_ + 3H_2_O → 3CO_2_ + 7H_2_ (7)

**FIGURE 4:**
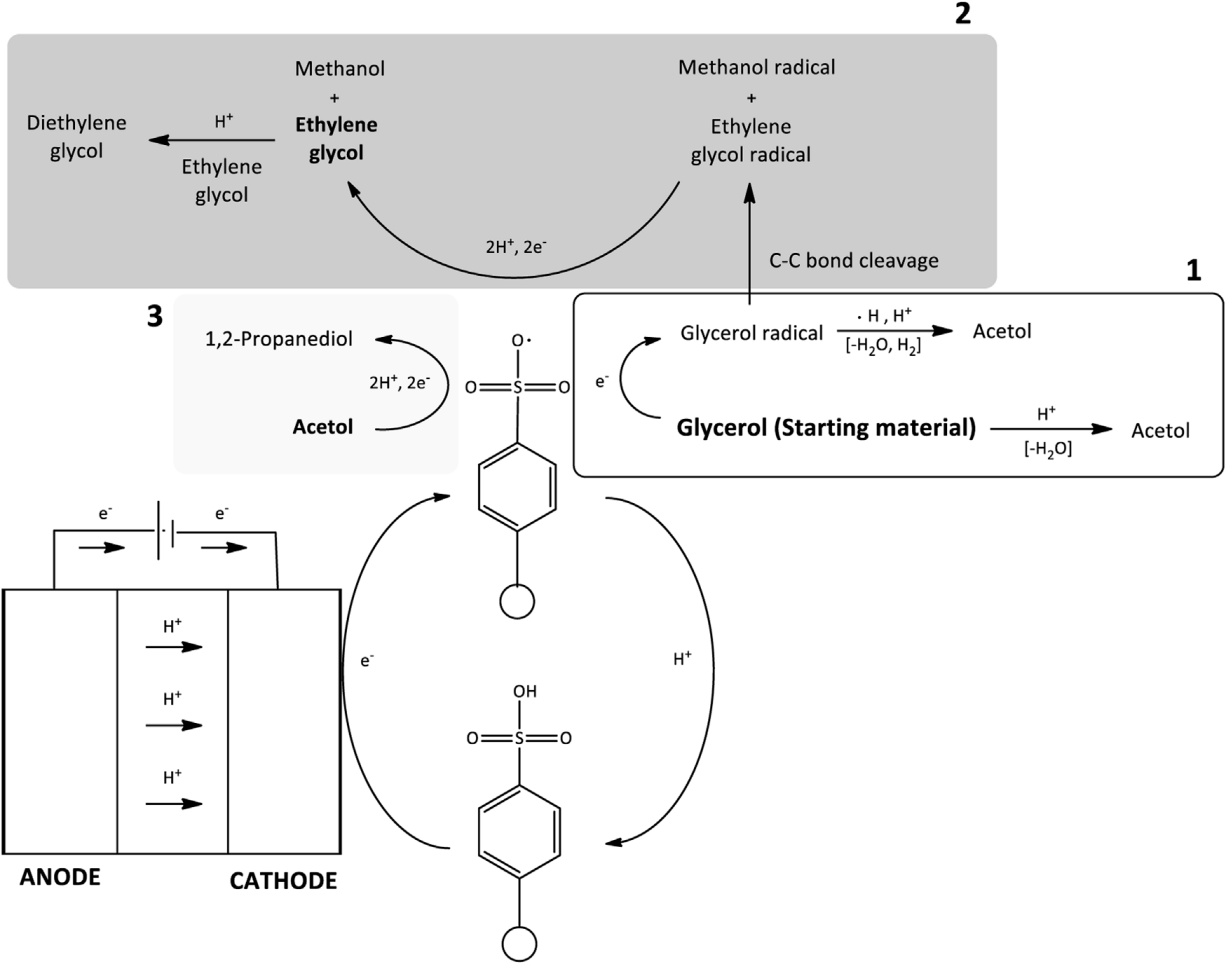
Overall identified reaction mechanisms of glycerol electrocatalytic reduction reaction.

However, this work established glycerol can also be reduced to other valuable products such as 1,2-propanediol and diethylene glycol with acetol and ethylene glycol as the crucial intermediates. Encapsulate to the products distribution results obtained from the mechanistic study, the overall reaction mechanisms have been identified referring the literature reports (Kongjao et al., 2011; Hunsom and Saila, 2015; Freitas et al., 2018; Yfanti et al., 2018), and the basics of electrochemistry (Steckhan, 1986; Francke and Little, 2014; Kai et al., 2017). Partial electrocatalytic reduction of glycerol involved multiple parallel and consecutive reactions, where the reduction products were founded from three possible mechanism pathways (Figure 4). According to this figure, the main pathways can be categorized into four types which are *1*) acid protonation and hydration, *2*) direct or indirect reduction with electricity, *3*) reduction with hydride radicals (H•) that are produced by the H^+^ ions adsorption on the electrode and *4*) isomerization of intermediates. Their first intermediary step is important to determine the production of 1,2-propanediol or diethylene glycol.

**SCHEME 2:**
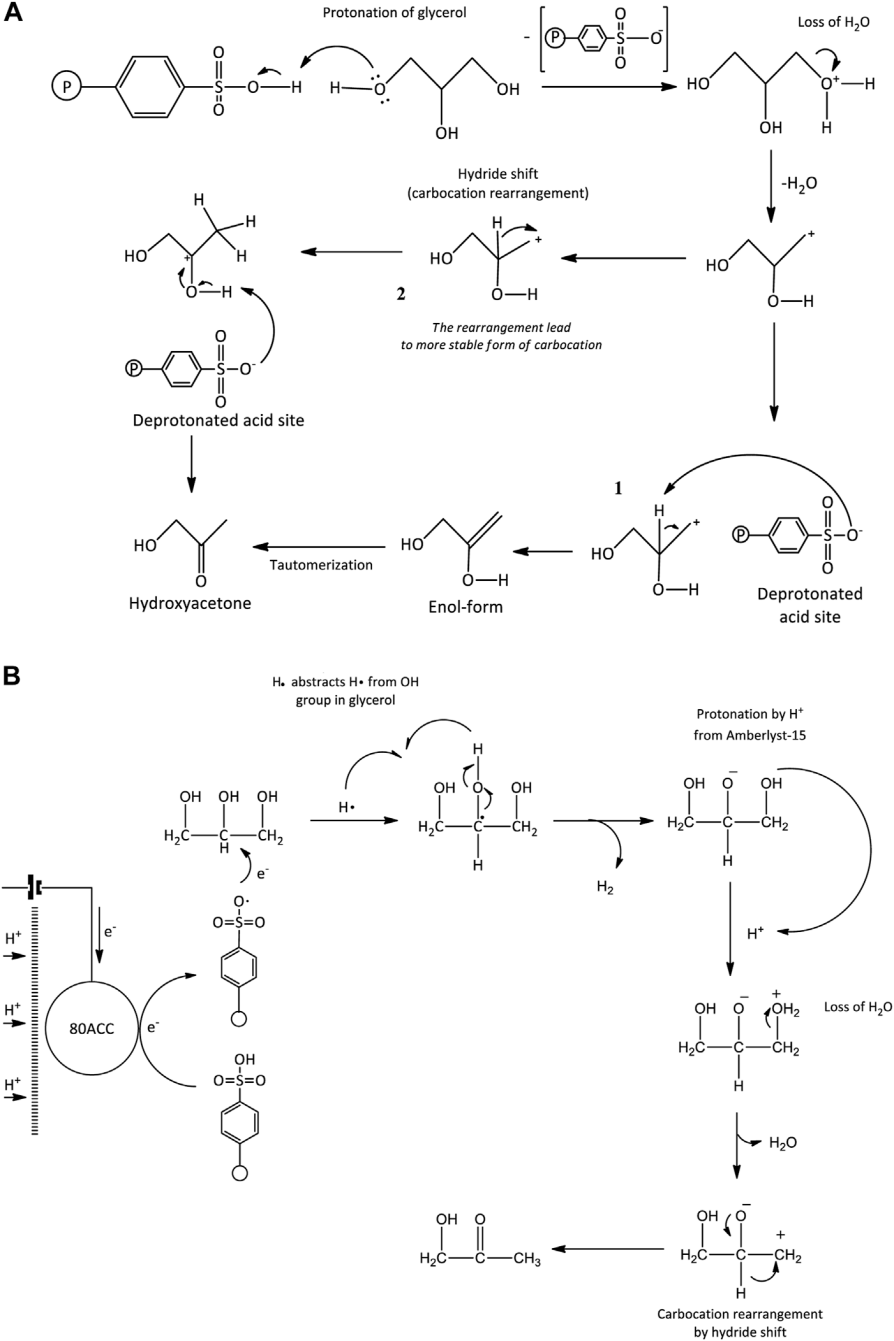
**(A)** Dehydration of glycerol via pinacol rearrangement and **(B)** reductive reaction mediated by Amberlyst-15 mechanism pathways.

Under the highly acidic condition, acetol was obtained through dehydration by water removal from glycerol molecule and *via* the C-O bond dissociation in its molecule. The detailed mechanism reaction is presented in Scheme 2. Glycerol dehydration into acetol infers one of the OH groups removals at the terminal carbons in glycerol molecule, whereas the acrolein formation includes the abstract of OH group from the central carbon through the unstable 3-hydroxypropenal. These routes are mostly controlled by the nature of the acid sites, and it is believed that Brønsted acid sites facilitate the selectivity towards acrolein while Lewis acid sites catalyze acetol production (Stošić et al., 2012; Célerier et al., 2018). In contrast, Amberlyst-15 has Bronsted acid sites (Pal et al., 2012; Cecilia et al., 2015), thus, the Lewis acid mechanism could not be applied. Additionally, Nimlos et al. (2006) found the transition state energy (E = 70.9 kcal/mol) for 1,2-dehydration in neutral glycerol through this mechanism is relatively high. This high energy barrier is more likely for reactions with high temperatures such as pyrolysis and combustion. The reaction mechanism is rather undergone a pinacol rearrangement or hydride transfer mechanism as shown in Scheme 2A. One of the OH groups was protonated by an H^+^ ion and a stable leaving group was established. There is water loss at the protonation site, resulting in carbocation in the glycerol molecule. A carbocation is known for its lack of electrons which creates it to be an overall positive charge on the carbon atom. Attributable to the OH groups’ position in glycerol, two carbocation intermediates can be produced. The first intermediate is carbocation that positioned at the terminal carbon atom. H atom bonded to the neighboring C atom was simultaneously removed by the deprotonated Amberlyst-15, forming an enol intermediate. Acetol was obtained through the tautomerization pathway from this intermediate. In the second intermediate, the carbocation that positioned at the terminal C atom rearranged (hydride shift) into carbocation in the middle chain to stabilize the carbon atom. Hydrogen atom was removed from OH group and regenerated the deprotonated Amberlyst-15. A stable double bond ketone (acetol) was finally developed.

**SCHEME 3:**
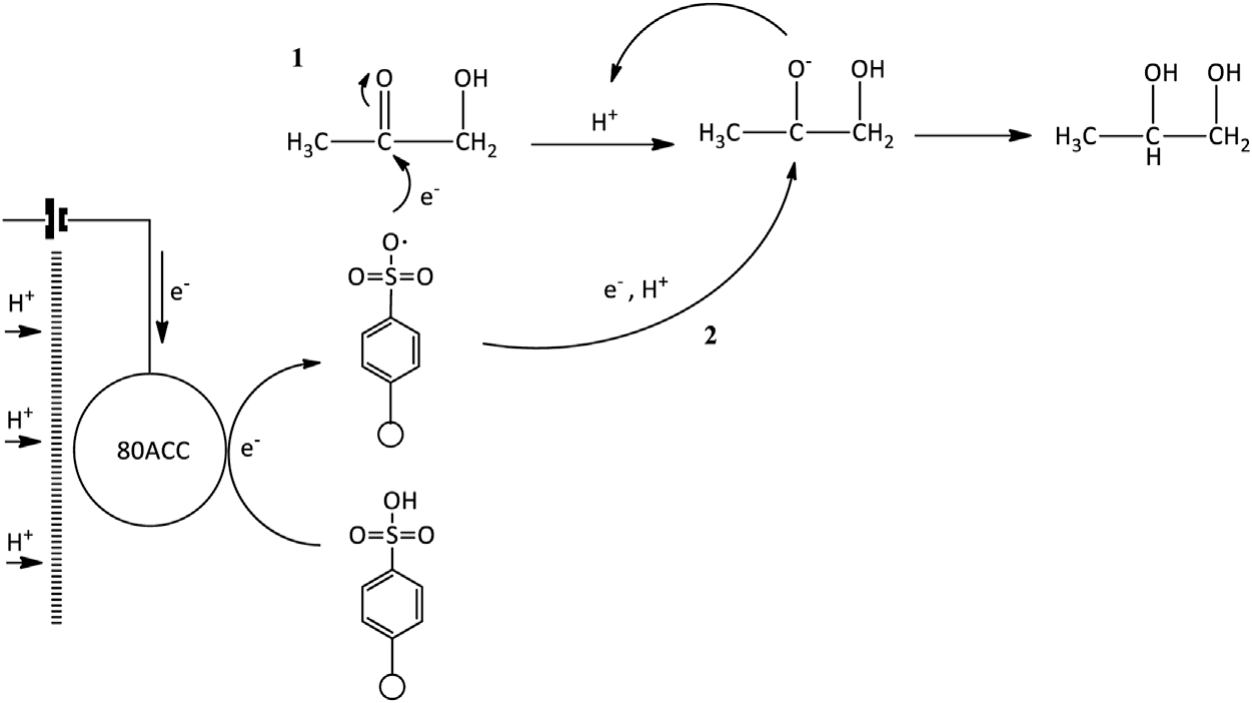
Electrocatalytic hydrogenation of acetol.

In the presence of the redox mediator, the formation of acetol from electrocatalytic reduction mediated by Amberlyst-15 can also happen and the reaction mechanism is presented in Scheme 2B. When the electricity was applied, the electrons transferred from the 80ACC electrode to Amberlyst-15 and further activated it into Amberlyst-15 radical anion (A-15•^–^). A single electron was then transmitted to glycerol and produced glycerol radical (CH_3_OHC•HOHCH_3_OH) (Steckhan, 1986). As hydrogen evolution reaction (HER) has simultaneously occurred at the cathodic region, H• radicals formed through *1*) Volmer-Heyrovsky or *2*) Volmer-Tafel mechanisms (Murthy et al., 2018; Nemiwal et al., 2021) can abstract H• atom at OH group of C_1_ or C_2_ position in glycerol radical molecule. The H• removal in the form of H_2_ happened with the excess of protons in the acidic medium which caused the protonation of the OH group. A stable leaving group was extracted as water. The hydride shift took place and subsequently, the intermediate was rapidly rearranged into acetol. Acetol with -C=O (carbonyl group) is a reactive species that was reduced into 1,2-propanediol through the electrocatalytic hydrogenation route. The simultaneous addition of protons (H^+^ ions) and electrons from the anode part through the activated Amberlyst-15 radical anion (A-15•^–^) managed to avoid over-reduction of glycerol into other minor products (Scheme 3).

**SCHEME 4:**
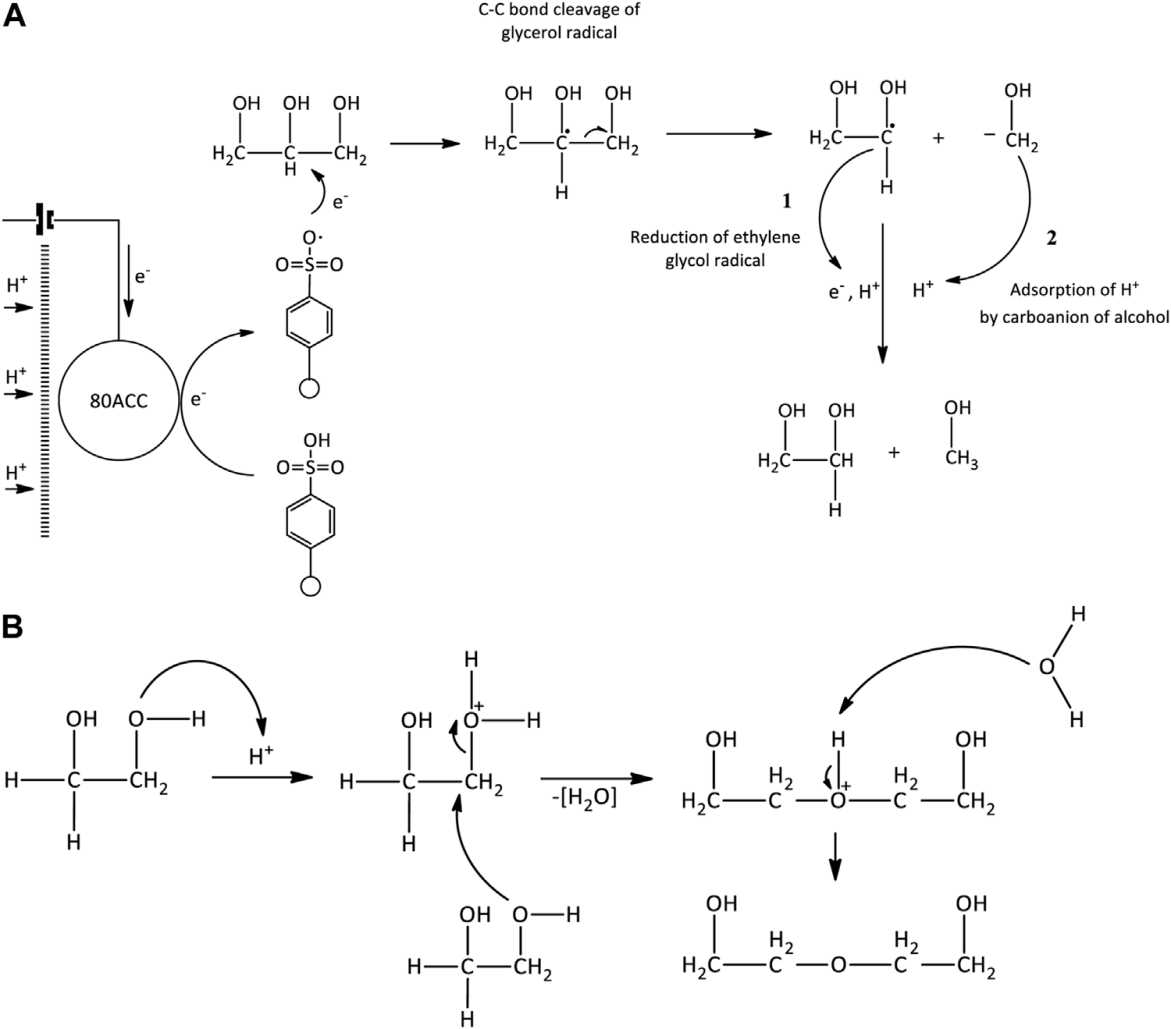
**(A)** Electrocatalytic cleavage of glycerol radical and **(B)** intermolecular dehydration of ethylene glycol.

Diethylene glycol was not obtained in acetol electrocatalytic reduction and glycerol experiment without electricity, suggesting that it was directly generated from glycerol by the presence of electrical current. From a mechanistic perspective, two free radical compounds formed through C-C bond cleavage in glycerol radical molecule is the initial step (Scheme 4A). Glycerol radical was dissociated into ethylene glycol radical and alcohol-free anion with the aid of Amberlyst-15 radical (A-15•^–^) (Steckhan, 1986). Ethylene glycol radical was reduced into ethylene glycol by a parallel route (electrocatalytic hydrogenation mechanism) in agreement with the earlier reports (Dieuzeide et al., 2017; Yfanti et al., 2018). In a highly acidic medium, intermolecular dehydration of ethylene glycol occurred and generated diethylene glycol where the route is identical to dipropylene glycol synthesis (Chitwood and Freure, 1946; Chiu et al., 2008; Yu et al., 2009). As shown in Scheme 4B, ethylene glycol was protonated by H^+^ ions and triggered the removal of water. At the same time, the OH group in the ethylene glycol with higher electrons affinity attacked the carbocation of another ethylene glycol. It was then rapidly rearranged into a stable form of diethylene glycol by the removal of H^+^ using H_2_O.

### Electrocatalytic Reduction of Glycerol to 1,2-Propanediol: Preliminary Experiments

Affixed to the mechanistic insight for glycerol electrocatalytic reduction in the previous section, the preliminary tests in terms of reaction temperature, initial concentration, and current density were accomplished using pure glycerol in Amberlyst-15 solution to find the best kinetics parameters condition for 1,2-propanediol. Figure 1B shows the chromatogram of the produced compounds after 8 h of reaction. 1,2-propanediol was the leading product with other minor compounds such as methanol, acetol, 3-methoxy-1,2-propanediol, 3-hydroxy-2-butanone, ethylene glycol, and diethylene glycol that were generated in small quantities.

#### Influence of Reaction Temperature

**FIGURE 5:**
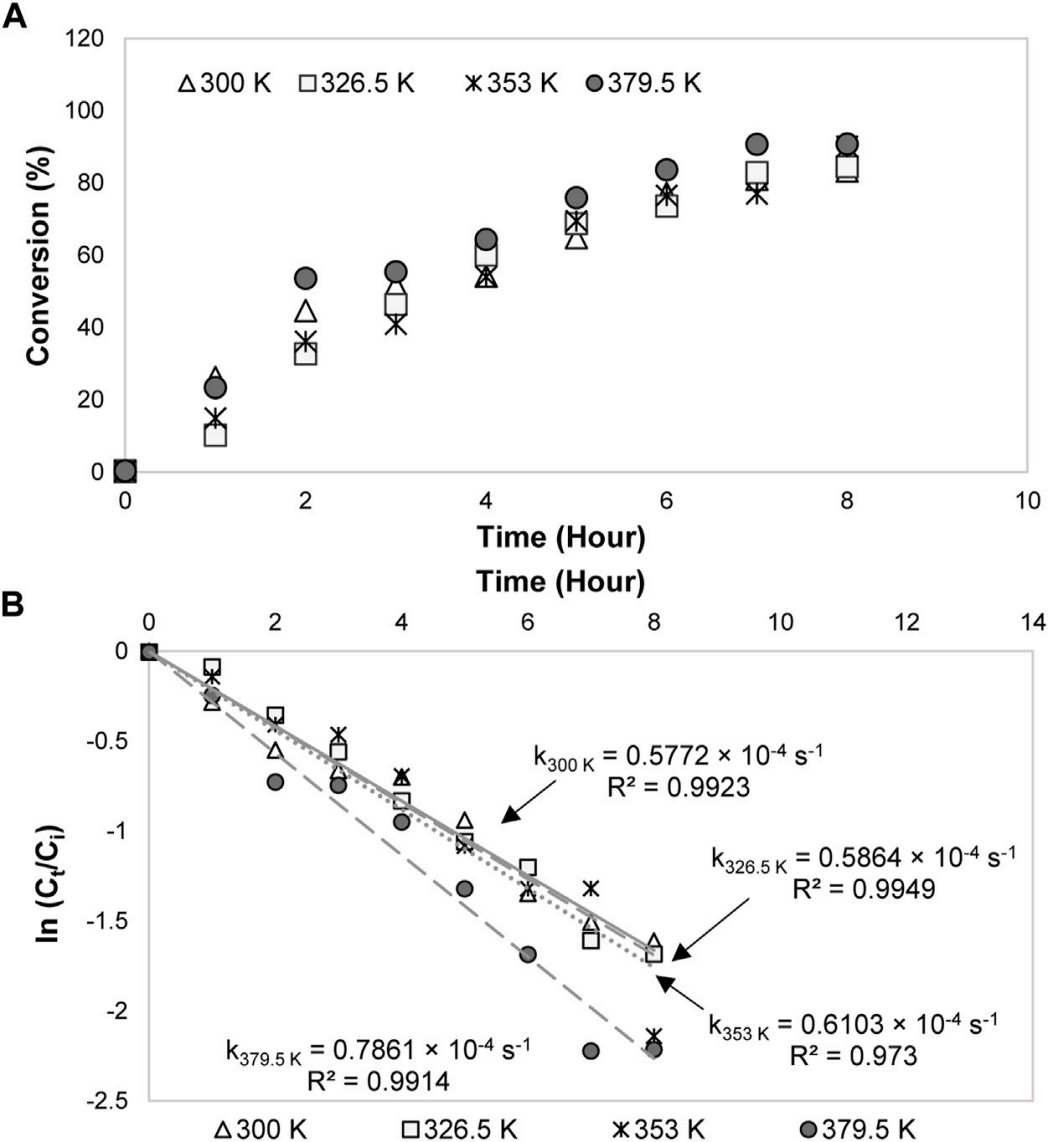
**(A)** Glycerol conversion and **(B)** first-order kinetics model for 0.3 mol/L of glycerol electrocatalytic reduction at different reaction temperatures.

Temperature variation on glycerol electrocatalytic reduction experiments was performed utilizing room temperature (300 K), 326.5, 353, and 379.5 K in 0.3 mol/L of glycerol and 9.6% (w/v) of Amberlyst-15 in sodium sulfate at 0.14 A/cm^2^ current density. The effect of reaction temperature on glycerol conversion and the kinetics rate constants is shown in Figure 5. The glycerol conversion was improved with the temperature rise. At low temperatures (300 and 326.5 K) the conversion of glycerol was similar around 84% (respective to the conversion rates of 0.5772 × 10^–4^ s^−1^ and 0.5864 × 10^–4^ s^−1^). It completed up to around 90% at high temperatures (353 and 379.5 K) and the kinetics rate constant reached the highest value of 0.7861 × 10^–4^ s^−1^ at the highest temperature (379.5 K). An increase in temperature has reduced the mixture viscosity and improved the glycerol diffusion process. It then enhanced the mass transfer of glycerol, promoting the interaction between its molecule or intermediates with Amberlyst-15 radical anion mediator (A-15•^–^) for the redox reaction with 80ACC electrode (Nascimento and Linares Leon, 2014; Lee et al., 2019). Consequently, it led to high glycerol conversion with more products. The reaction rate order in these experiments was similar to the previous work in the literature (Hunsom and Saila, 2013). In the H_2_SO_4_ electrolyte, the authors obtained kinetics rate constant of 0.4917 × 10^–4^ s^−1^ for glycerol electrochemical conversion, which was lower than our work. It signifies the presence of Amberlyst-15 able to accelerate the conversion of glycerol to value-added products.

**FIGURE 6:**
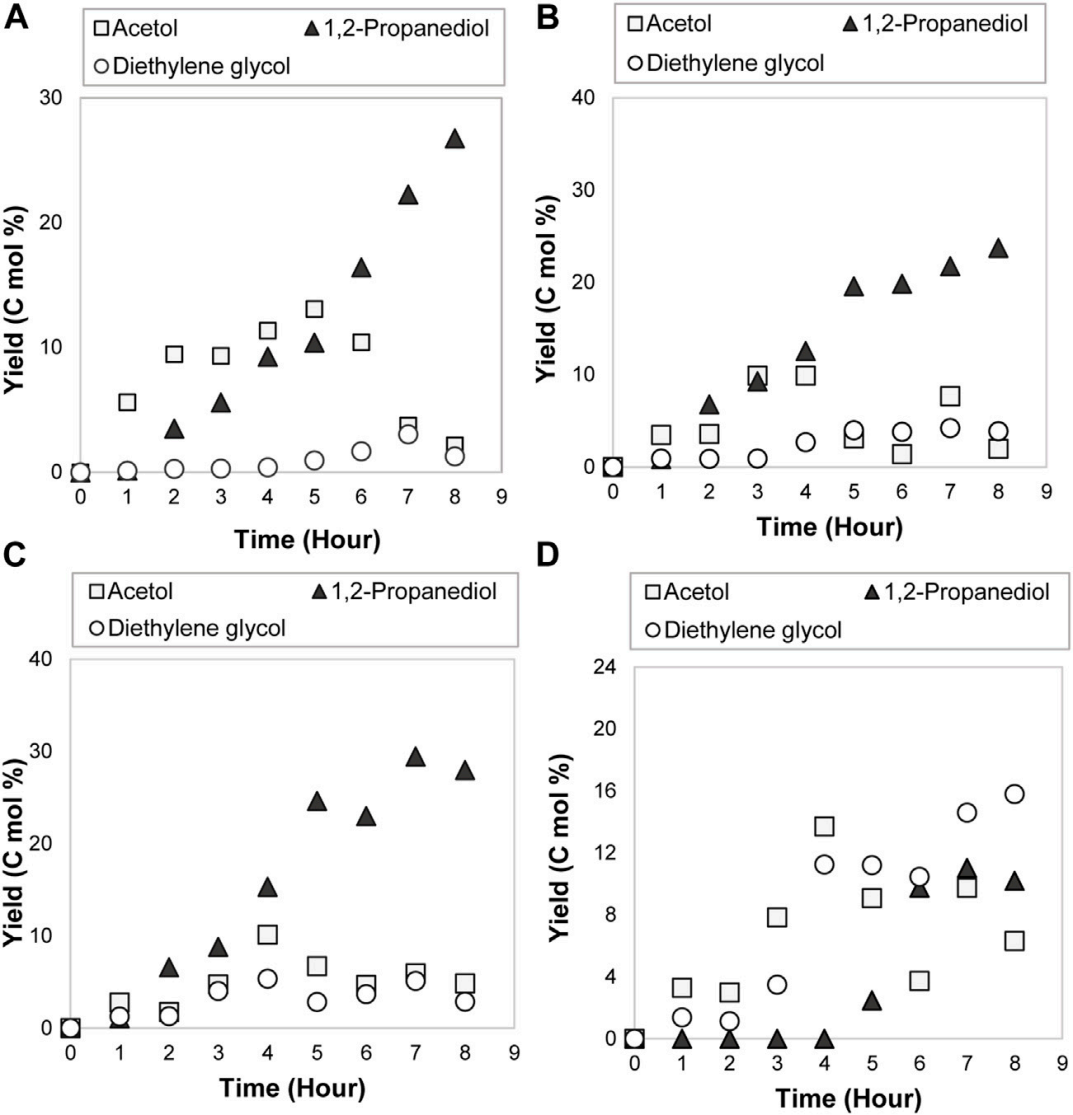
Products distribution for 0.3 mol/L of glycerol electrocatalytic reduction at **(A)** 300 K, **(B)** 326.5 K, **(C)** 353 K and **(D)** 379.5 K with 0.14 A/cm^2^ of current density.

During the reaction, a temperature improvement for long hours boosted both the C-O and C-C bonds breakage, transforming glycerol into acetol and diethylene glycol that came from ethylene glycol. From Figure 6, the maximum yield of acetol and diethylene glycol were 13.7 C mol% (fourth hour) and 15.9 C mol% (eighth hour) at 379.5 K. The fast dissociation was contributed by the large energy collected from high temperature, thereby, improved the molecular collisions frequency between electrolyte ions (Licona et al., 2014). The high number of molecular collisions speeded up the electrons passage between A-15•^–^ and 80ACC electrode in producing the intermediates and 1,2-propanediol during the redox reaction with glycerol. Moreover, the highest yield of diethylene glycol accomplished at this temperature demonstrated that ethylene glycol dehydration also favored a high temperature. Acetol and diethylene glycol yield kept increasing due to the incomplete glycerol electrocatalytic reduction reaction. Thus, shortening the electrolysis time can lessen glycerol interaction with A-15•^–^ and stop these undesirable compounds generation. Likewise, greater ionic conductivity and lower resistance at elevated temperature can increase the electrocatalytic hydrogenation of acetol towards the 1,2-propanediol formation. The yield was enhanced from 26.7 C mol% (300 K at the eighth hour) to 29.4 C mol% (353 K at the seventh hour). Albeit the high temperature is needed for 1,2-propanediol formation, it requires to mention that an additional escalation to higher temperature is not recommended. It can cause water evaporation in the solution and obstruct the reaction (Nascimento and Linares Leon, 2014). The compounds with a low boiling point like acetol might also vaporize. It can be seen in Figure 6D; acetol yield was spotted inconsistently at 379.5 K. As stated in the acetol experiments’ discussion, hydrogen evolution reaction (HER) can be the primary reaction at low substrate concentration. H^+^ ions in the aqueous solution combated for hydrogen (H_2_) production and acetol hydrogenation reaction into 1,2-propanediol. At too high temperature, H^+^ ions and electrons that were transferred from the anode part were reduced into hydrogen quicker than acetol hydrogenation reaction. The developed H_2_ gases on the cathode electrode surface can be a carrier agent for highly volatile compounds like acetone, methanol, and acetol. These minor products are expected to purge out with hydrogen in a significant yield. Indeed, the losses can be abated by an appropriately sealed reactor setup and the prevention of excessive HER. From Figure 6, a suitable temperature range for a selective C-O bond cleavage into acetol and successive hydrogenation to 1,2-propanediol reaction were the low or moderate temperature. Yet, 353 K temperature produced the greatest yield of 1,2-propanediol, which was then used in the subsequent operating parameters evaluation.

#### Influence of Glycerol Initial Concentration

**FIGURE 7:**
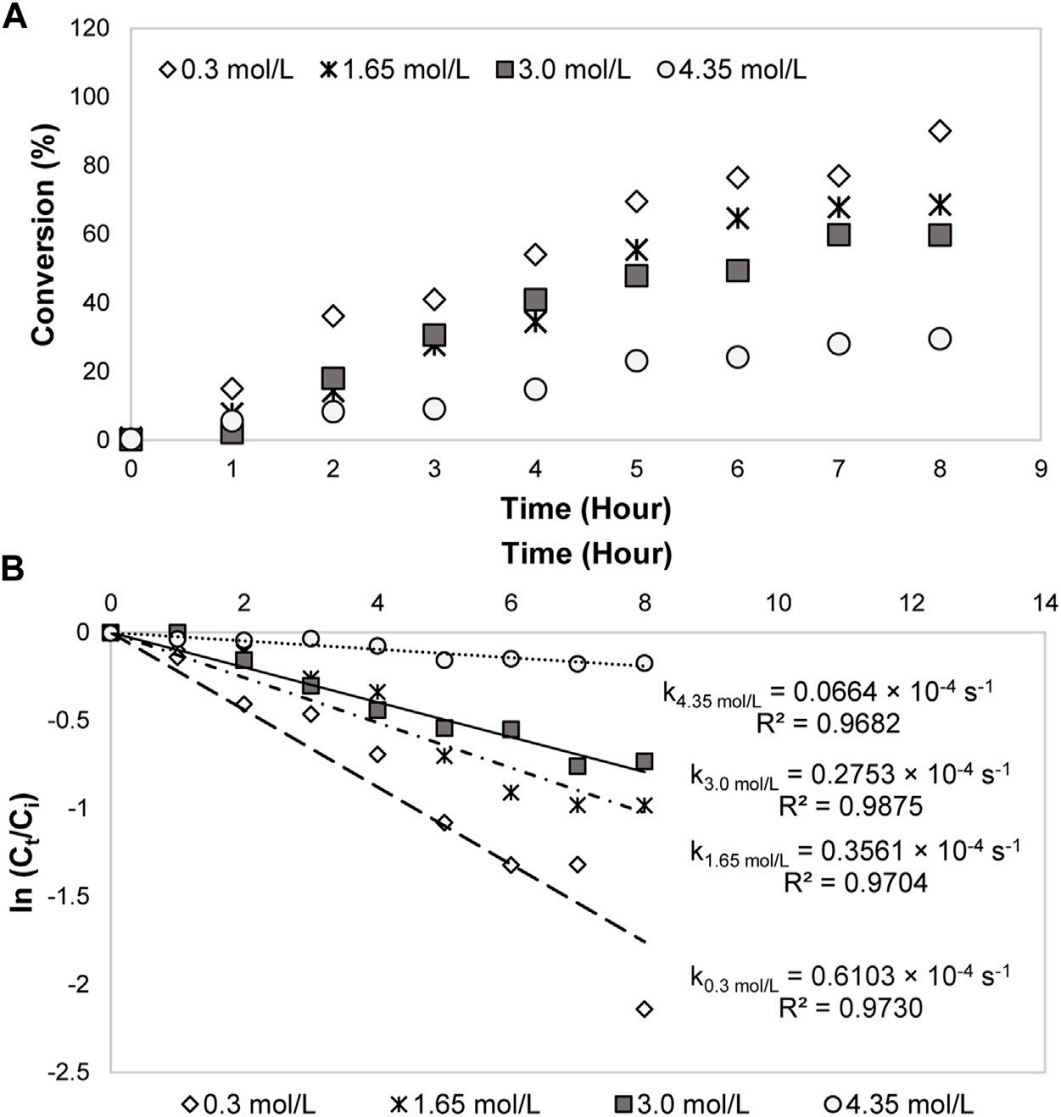
**(A)** Glycerol conversion and **(B)** first-order kinetics model for electrocatalytic reduction of glycerol with different initial concentrations.

0.3, 1.65, 3.0, and 4.35 mol/L of pure glycerol were explored at a constant temperature (353 K) and current density (0.14 A/cm^2^) to ascertain the effect on the reaction mechanisms and products distribution. From Figure 7A, after 8 h of reaction, the glycerol conversion was reduced from 90% (0.3 mol/L) to 30% (4.35 mol/L) when the initial concentration was increased. The decline was because of the higher viscosity in the concentrated glycerol. Support by Nascimento and Linares Leon (2014) work, at high glycerol concentration, it was found that too high viscosity of glycerol limited the molecule transport to the electrocatalytic layer, causing the anode surface poisoning. The phenomenon occurred because of the large amounts of glycerol competing with the hydroxyl radicals for the electro-oxidation reaction. In this work, the cathode electrode efficiency was inhibited due to a similar reason. Too high glycerol concentration reduced the mass transport of glycerol molecule for the reaction with 80ACC electrode through A1-5•^–^, leading to a slow conversion. Based on first-order kinetics plots (Figure 7B), the kinetics rate constant of 0.3 mol/L glycerol was the fastest (0.6103 × 10^–4^ s^−1^), followed by 1.65, 3.0 and 4.35 mol/L concentrations (0.3561 × 10^–4^ s^−1^, 0.2753 × 10^–4^ s^−1^ and 0.0664 × 10^–4^ s^−1^ respectively).

**FIGURE 8:**
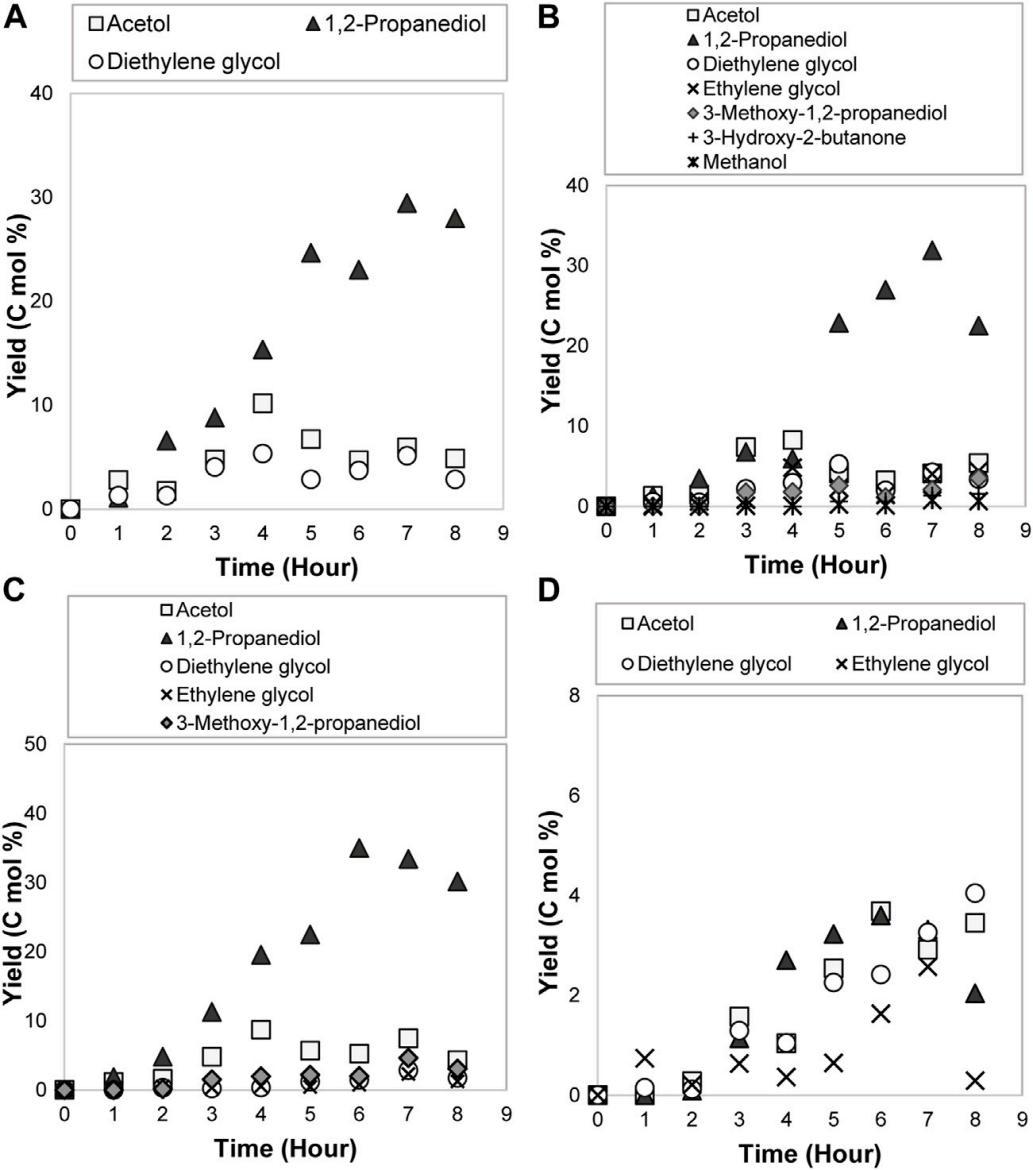
Products distribution with glycerol initial concentration of **(A)** 0.3 mol/L, **(B)** 1.65 mol/L **(C)** 3.0 mol/L and **(D)** 4.35 mol/L during the electrocatalytic reduction reaction at 353 K and 0.14 A/cm^2^.

From Figure 8, 1,2-propanediol was the major compound for 0.3, 1.65, and 3.0 mol/L concentrations. The greatest yield was attained at the seventh hour for 0.3 mol/L (29.4 C mol%) and 1.65 mol/L (31.9 C mol%), sixth hour for 3.0 mol/L (35.0 C mol%). There is no direct time-dependence for 1,2-propanediol yield because it may produce other by-products with high volatility such as acetone. It was previously discussed that acetone was only observed below 353 K temperature. Substantial to other new products such as methanol, ethylene glycol, 3-methoxy-1,2-propanediol, and 3-hydroxy-2-butanone appeared at 1.65 and 3.0 mol/L, the reaction mechanisms were confirmed where glycerol can undergo the C-C bond dissociation to methanol and ethylene glycol in the presence of electricity. Methanol obtained from the C-C bond cleavage of glycerol reacted with the unconverted glycerol and produced 3-methoxy-1,2-propanediol through an etherification process. The hydroxide ion (OH^−^) was abstracted from methanol whereas one proton was removed from glycerol, which was catalyzed by Amberlyst-15 catalyst (Pico et al., 2012). This mechanism developed 3-methoxy-1,2-propanediol and water as a by-product. 3-hydroxy-2-butanone was formed from acetol where its formation was favored at higher concentration. Nonetheless, at 4.35 mol/L of glycerol, the products combated with glycerol reactant for the redox reaction with A-15•^–^, consequently triggering a self-inhibition towards the total yield of products (Farma et al., 2013). This implies that glycerol conversion to 1,2-propanediol and intermediates was not favorable at the highest concentration. In contrast, at a minimum and medium concentration, more 1,2-propanediol with C_5_ to C_2_ products were produced. Additionally, acetol has been identified as the main intermediate product with a yield slightly higher than ethylene glycol and diethylene glycol. The yield remained approximately 10.0 C mol% for each concentration, demonstrating the reduction of acetol to 1,2-propanediol is a fast-consecutive reaction. In general, although glycerol was not completely converted, 3.0 mol/L of acetol was sufficient to improve the yield of 1,2-propanediol. Hence, this concentration was applied in the following study.

#### Influence of Current Density

**FIGURE 9:**
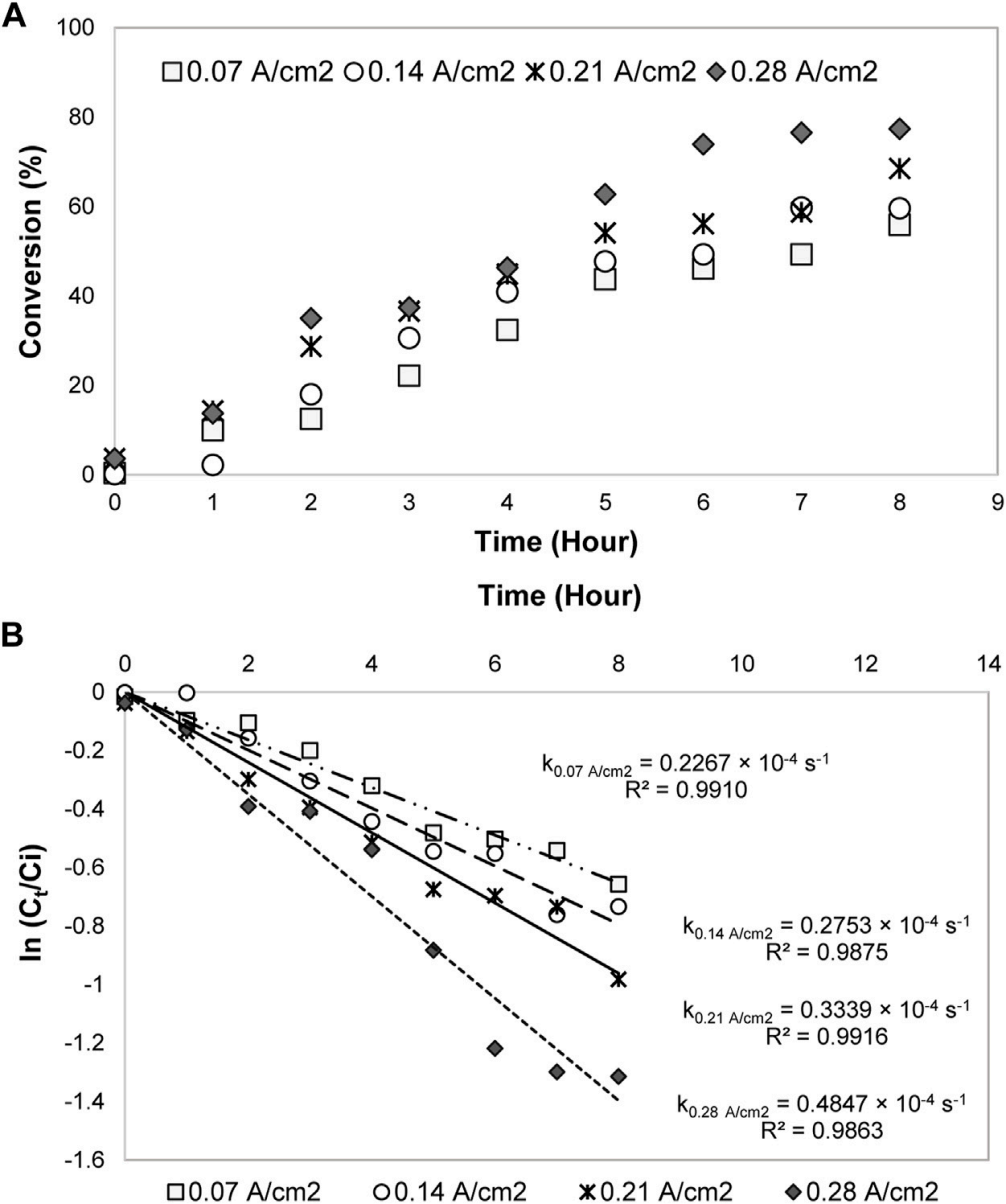
**(A)** Glycerol conversion (%) and **(B)** first-order kinetics model for glycerol electrocatalytic reduction at different current densities.

After the optimum temperature and glycerol concentration were achieved, the electrical current was varied from 0.05 to 1.0, 1.5, and 2.0 A (equivalent to 0.07, 0.14, 0.21, and 0.28 A/cm^2^ of current densities). As shown in Figure 9A, glycerol conversion was increased with the current density enhancement from 0.07 A/cm^2^ (56%) to 0.28 A/cm^2^ (76%) after 8 h. A complete conversion may take a prolonged time for a lower current density. Compatible with Faraday’s law, the conversion rate was improved from 0.2267 × 10^–4^ s^−1^ (0.07 A/cm^2^) to 0.4847 × 10^–4^ s^−1^ (0.28 A/cm^2^) (Figure 9B).

**FIGURE 10:**
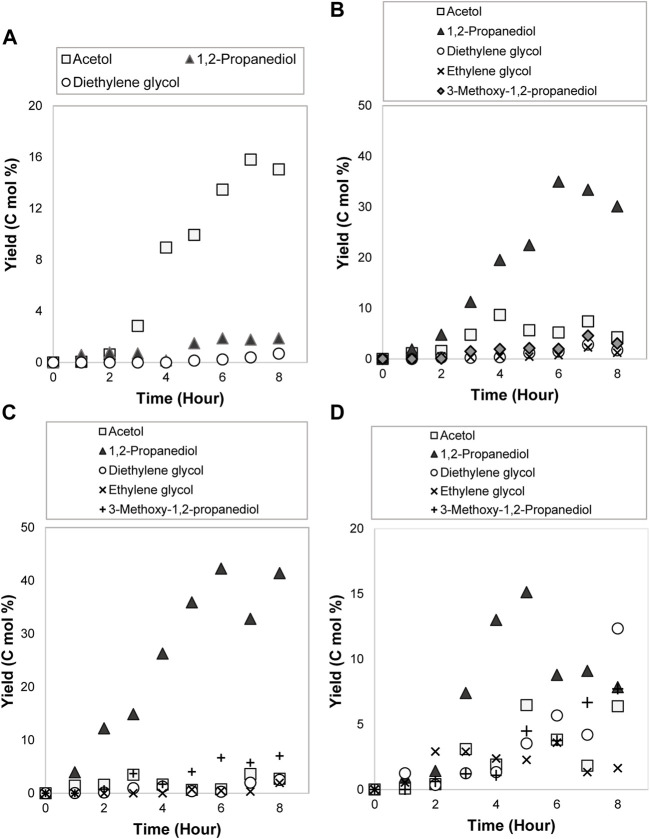
Products distribution at **(A)** 0.07 A/cm^2^, **(B)** 0.14 A/cm^2^, **(C)** 0.21 A/cm^2^, and **(D)** 0.28 A/cm^2^ of current densities during 3.0 mol/L of glycerol electrocatalytic reduction at 353 K.

Related to the impact on the products distribution (Figure 10), acetol is the major compound (with 15.8 C mol%, seventh hour) generated at 0.07 A/cm^2^. It suggests the C-O bond breakage of glycerol preferred low current density and its development is the earliest step in glycerol electrocatalytic reduction reaction. The small amount of 1,2-propanediol suggested the sluggishness of acetol electrocatalytic hydrogenation at this current density. For high 1,2-propanediol yield, medium to high current densities (0.14–0.21 A/cm^2^) showed the best result with the maximum yield of 42.3 C mol % (sixth hour) at 0.21 A/cm^2^. The greater the current density, the faster acetol electrocatalytic hydrogenation reaction to 1,2-propanediol. This is because more electrons and protons were provided from the anodic compartment for this reaction. However, the yield of 1,2-propanediol was reduced to 15.1 C mol% at the sixth hour with the largest current density. Higher electrical current can trigger the fragmentation of glycerol or 1,2-propanediol to gases products, which cannot be detected in the liquid phase analysis. HER may as well prefer a high electrical current since more electrons were accessible in the process. Therefore, medium current density is excellent for a selective and high yield for 1,2-propanediol. Whereas ethylene glycol was only detected at medium to high current density. In conformity to higher electrode potential enhanced the C-C bond cleavage by Colmati et al. (2009); Gomes and Tremiliosi-Filho (2011), higher current density also led to higher production of ethylene glycol through C-C bond breakage of glycerol. The rapid rate of ethylene glycol dehydration was facilitated by a high current same as the etherification of glycerol with methanol. Diethylene glycol and 3-methoxy-1,2-propanediol accomplished 9.3 C mol% and 5.8 C mol% yields, respectively at 0.28 A/cm^2^. A high current density can promote the conversion of glycerol into various valuable compounds especially, 1,2-propanediol. Nevertheless, too high current density does not develop the yield of the targeted compound. Indeed, it initiated the formation of unwanted gases products and removed the minor products with high volatility.

To conclude, the initial step involved the C-O and C-C bonds cleavage in glycerol play a crucial role in producing either acetol or ethylene glycol intermediate. This was controlled by the temperature, where low to medium value is needed to maintain a selective acetol-1,2-propanediol route. In addition, moderate concentration reduced the hydrogen formation and indirectly improved the 1,2-propanediol yield. A mild current density raised the conversion rate and minimized the intermediates growth. Although the greatest conversion of glycerol (76%) was achieved at 0.28 A/cm^2^, however, the operating condition of 353 K reaction temperature, 3.0 mol/L glycerol initial concentration at 0.21 A/cm^2^ was found to be optimum for 1,2-propanediol production. This is because this condition reached the maximum yield (42.3 C mol%) and selectivity (75%) at a quicker time (at the sixth hour) compared to other conditions.

### Energy Consumptions for Acetol and Glycerol Electrocatalytic Reduction Reaction

The elementary steps for each reaction that were discussed in the reaction mechanisms section are described in Table 2. Normally, taken from stoichiometry Eq. 6, 14 electrons and 14 protons are needed to yield 7 mol of hydrogen in HER. However, to transform each mole of glycerol into valuable products like acetol, one proton or 1 H• atom, one proton and one electron for acetol dehydration and electrocatalytic reductive reactions (step 1 and 2, respectively). Two electrons and two protons for 1,2-propanediol from acetol (step 3), two electrons and two protons for ethylene glycol from glycerol through the indirect reaction (step 4). Meanwhile, each 1 H^+^ is involved for every diethylene glycol (Step 5) and 3-methoxy-1,2-propanediol (Step 6) during the intermolecular dehydration and etherification reactions. The first-order kinetics model was found to be the best for all separate reactions’ experiments (Step 1, step 3, and step 5). The kinetics rate constants (*k*) from the model and energy consumptions are tabulated in Table 2.

**TABLE 2:** Elementary steps for the reactions.

Steps	Operating conditions^a^	Elementary steps for reaction mechanisms	k^a^ (s^−1)^	W (kWh/kg)
1st step (Glycerol dehydration)	[Gly] = 3.0 mol/L	C_3_H_8_O_3_ + H^+^ → (C_3_H_9_O_3_)^+^ → C_3_H_6_O_2_ + H^+^ + H_2_O	$\left(k_{C3H6O2}\right)$	—
	T = 353 K		k_1_ = 0.0436 × 10^–4^	
2nd step (Electrocatalytic reduction of glycerol) with H_2_ formation		C_3_H_8_O_3_ + e^−^ → (C_3_H_9_O_3_)•^–^	$\left(k_{{\left(C3H9O3\right)}^{•-}}\right)$	—
		(C_3_H_9_O_3_)•^-^ + H• + H^+^ + e^−^ → C_3_H_6_O_2_ + H_2_O + H_2_	$\left(k_{C3H6O2}\right)$	—
		H^+^ + e^−^ → ACC-H_ads_	$\left(k_{H•}\right)$ _Volmer_	—
		ACC-H_ads_ + H^+^ + e^−^ → H_2_ + ACC	$\left(k_{H2}\right)$ _Heyrovsky_	—
		ACC-H_ads_ + ACC-H_ads_ → H_2_ + 2ACC	$\left(k_{H2}\right)$ _Tafel_	—
3rd step (Electrocatalytic hydrogenation of acetol)	[ACTL] = 3.0 mol/L	C_3_H_6_O_2_ + 2H^+^ + 2e^−^ → C_3_H_8_O_2_	$\left(k_{C3H8O2}\right)$	10.17
	T = 353 K			
	*j* = 0.28 A/cm^2^		k_2_ = 0.4892 × 10^–4^	
	E = 25.7 V			
4th step (Electrocatalytic reduction and hydrogenation of glycerol)		C_3_H_8_O_3_ + e^−^ → (C_3_H_9_O_3_)•^–^	$\left(k_{{\left(C3H9O3\right)}^{•-}}\right)$	—
		(C_3_H_9_O_3_)•^-^ → C_2_H_5_O_2_• + CH_2_O^−^	$\left(k_{C2H5O2•\;}\right)$	—
		C_2_H_5_O_2_• + H^+^ + e^−^ → C_2_H_6_O_2_	$\left(k_{C2H6O2}\right)$	—
		CH_2_O^−^ + H^+^ → CH_3_O	$\left(k_{\mathrm{CH}3O}\right)$	—
5th step (Dehydration of ethylene glycol to diethylene glycol)	[EG] = 3.0 mol/L	C_2_H_6_O_2_ + H^+^ → (C_2_H_7_O_2_)^+^ + C_2_H_6_O_2_ → C_4_H_10_O_3_ + H^+^ + H_2_O	$\left(k_{C4H10O3}\right)$	—
	T = 353 K		k_3_ = 0.0867 × 10^–4^	
6th step (Etherification of glycerol with methanol)		C_3_H_8_O_3_ + CH_3_OH → C_4_H_10_O_3_ + H_2_O	$\left(k_{C4H10O3}\right)$	—
Overall (Glycerol electro-reduction reaction)	[Gly] = 3.0 mol/L	C_3_H_8_O_3_ + H^+^ +e^−^ → C_3_H_6_O_2_ + C_3_H_8_O_2_ + C_4_H_10_O_3_	k = 0.3339 × 10^–4^	5.24
	T = 353 K			
	*j* = 0.21 A/cm^2^			
	E = 21.9 V			

a At the optimal conditions for targeted compound formation; ACC, active site of 80ACC electrode; k, kinetics rate constant; W, electrical energy or energy consumption.

Energy consumptions in the processes were calculated using Eq. 6 relied on the kinetics parameters used during the reactions. *W* is energy consumed in glycerol (or acetol) conversion (kWh/kg), *I* is the current (A), *E* is the voltage (V) and *C*_*0*_ is the initial concentration (g/L), *C*_*t*_ is the final concentration (mol/L), *V* is the volume (L), and *M* is the molecular weight of compound.

$$W_{Gly\left(or\;acetol\right)conversion}\frac{IE\Delta t}{\left(C_{0}-C_{t}\right)VM}$$ (6)

Although the conversion rate for acetol electrocatalytic hydrogenation was better than glycerol electrocatalytic reduction, the consumed energy was doubly higher (10.17 kWh/kg) than the latter reaction (5.23 kWh/kg). This is due to the required voltage to generate 1,2-propanediol using acetol being larger than for glycerol reaction.

## Conclusion

In summary, the mechanistic experiments validated acetol was the major intermediate for 1,2-propanediol while the intermediate for diethylene glycol was ethylene glycol. Glycerol was also tested for the dehydration reaction and acetol was found as the main product, proving the necessity of H^+^ ions for a selective acetol-1,2-propanediol formation in the Amberlyst-15 solution. Optimal conditions reported in this work changed depending on the types of feedstocks and types of reactors. The ideal condition for acetol electrocatalytic hydrogenation in a two-compartment reactor was 3.0 mol/L initial concentration at 353 K and 0.28 A/cm^2^ (7 h of electrolysis) to generate the highest yield (59.8 C mol%) and selectivity (77%) of 1,2-propanediol. This condition offered the best energy consumption of 10.17 kWh/kg for acetol as the intermediate platform molecule. Whereas the optimized condition for glycerol electrocatalytic reduction into 1,2-propanediol able to doubly decrease the energy consumption and obtain the yield of 42.3 C mol% (75% selectivity) at a quickest time (sixth hour). This finding shows the excellent results among the published reports on the electrochemical conversion of glycerol into 1,2-propanediol. To sum up, the data obtained by this research will empower the possibility of 1,2-propanediol production from biomass-derivative glycerol to be done using the inexpensive and simple electrolysis technique. Certainly, it will open more research opportunities towards applying the activated carbon-based electrode for the electrochemical reactions in the electro-organic synthesis. Nevertheless, this practice still demands more investigation and improvement on the separation techniques to reliably deliver a greater purity (selectivity) of any product which are operational and economical for industrial needs. Evaluation of the concurrent effect and the significance of different operating kinetics parameters through response surface methodology (RSM) are essential and will be conducted in the further effort.

## Data Availability

The original contributions presented in the study are included in the article/Supplementary Material, further inquiries can be directed to the corresponding authors.
