# Supplementary material for: Glycerol Electrocatalytic Reduction Using an Activated Carbon Composite Electrode: Understanding the Reaction Mechanisms and an Optimization Study
Source: Front Chem. 2022 Feb 25;10:845614. doi: 10.3389/fchem.2022.845614 (PMC8914049; doi:10.3389/fchem.2022.845614)
Supplement: Supplementary file 1 [file DataSheet1.docx]

Supplementary Material

# Supplementary Figures and Tables

The supplementary figures consist of the products distribution and selectivity for the optimized condition. The MS spectra of presented compounds during the acetol and glycerol electrocatalytic reduction reactions as well as, glycerol and ethylene glycol experiments are also illustrated in this section. The figures of MS spectra have been modified for a better understanding.

## Supplementary Figures

**B**

**A**

Supplementary Figure 1: (A) Products distribution and (B) selectivity of acetol electrocatalytic reduction at the optimum conditions.


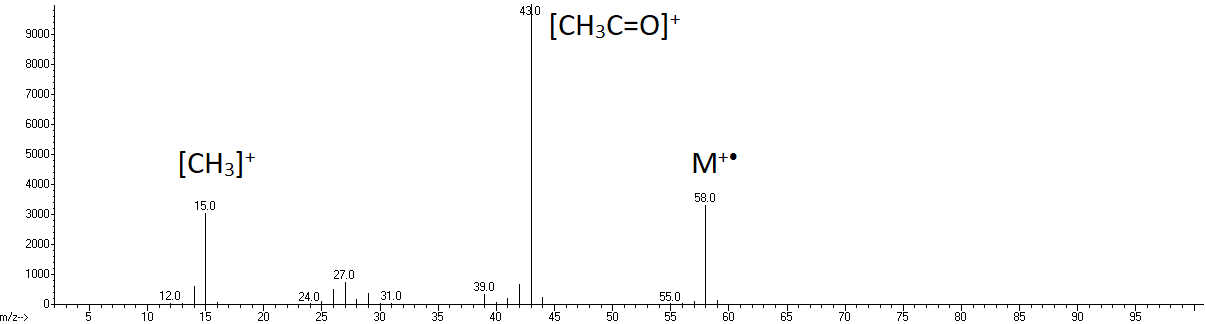

Supplementary Figure 2: MS spectrum for acetone at retention time of 2.24 min.

**
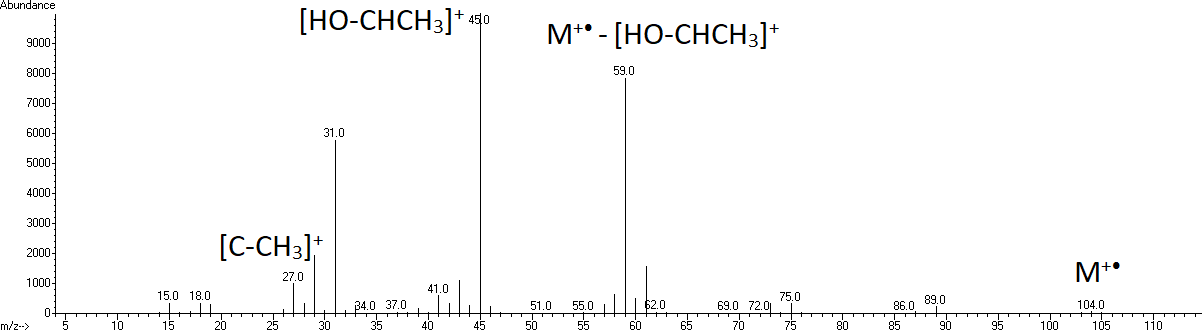
**

Supplementary Figure 3: MS spectrum for 1-ethoxy-2-propanol at retention time of 6.72 min.


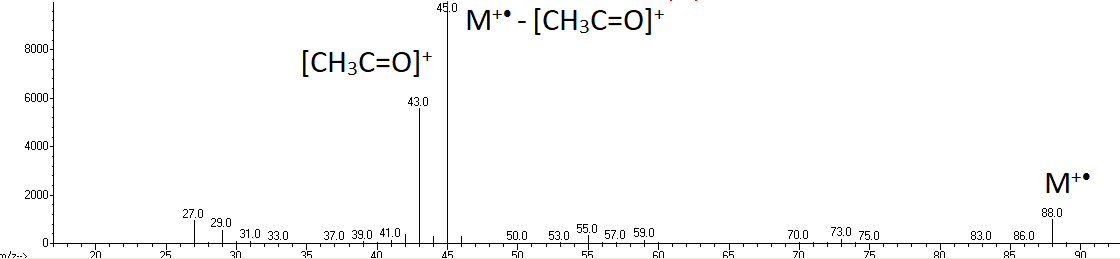

Supplementary Figure 4: MS spectrum for 3-hydroxy-2-butanone at retention time of 10.80 min.


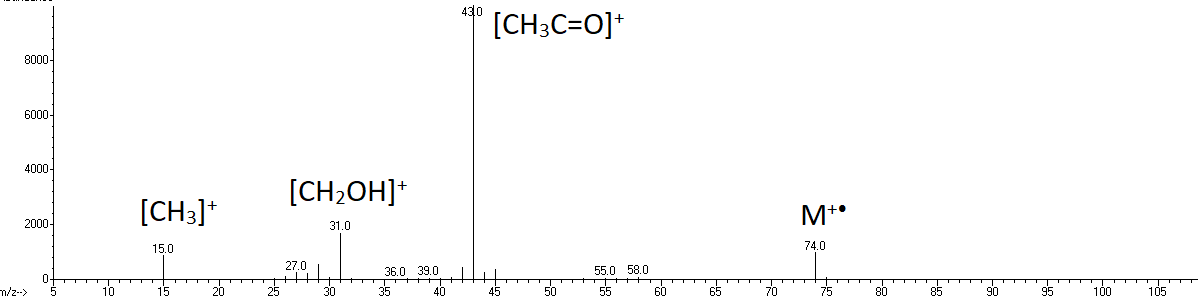

Supplementary Figure 5: MS spectrum for acetol at retention time of 11.2 min.


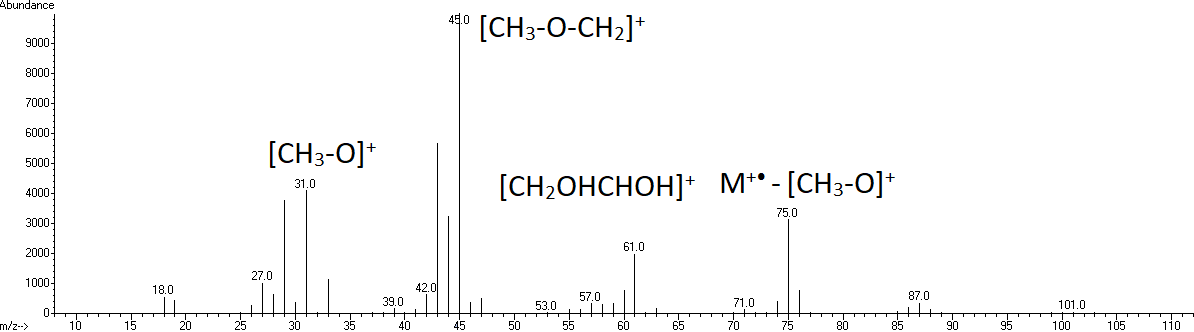

Supplementary Figure 6: MS spectrum for 3-methoxy-1,2-propanediol at retention time of 11.92 min.


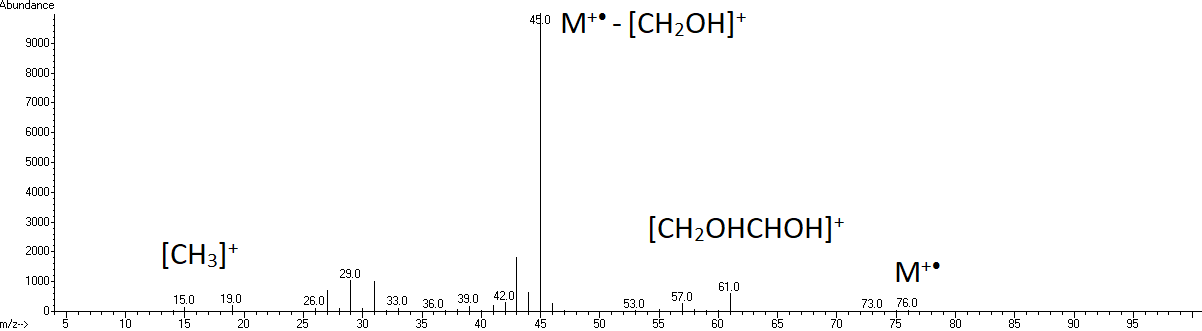

Supplementary Figure 7: MS spectrum for 1,2-propanediol at retention time of 14.75 min.


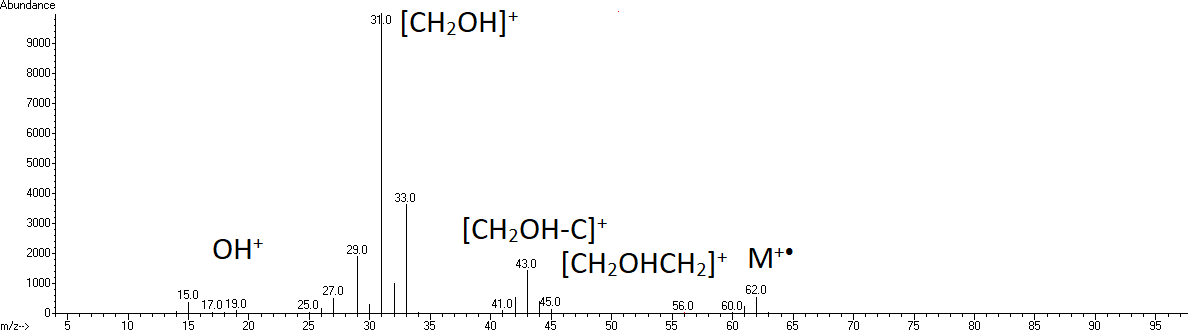

Supplementary Figure 8: MS spectrum for ethylene glycol at retention time of 15.32 min.


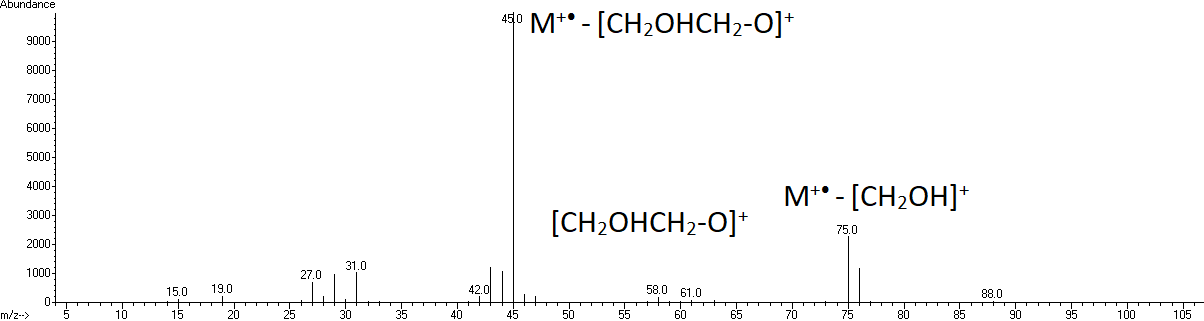

Supplementary Figure 9: MS spectrum for diethylene at retention time of 18.90 min.

**
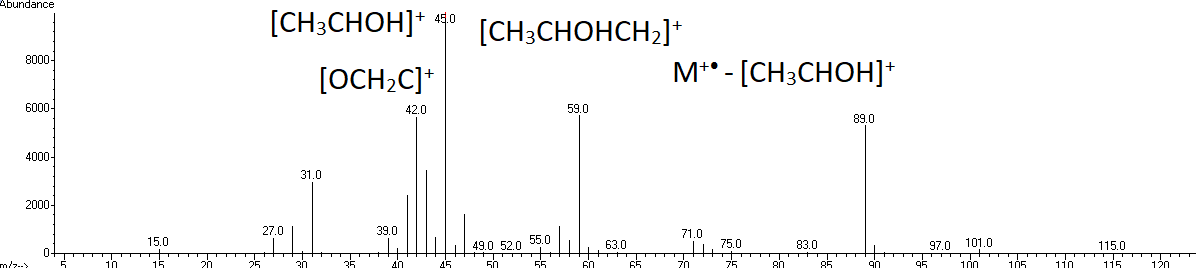
**

Supplementary Figure 10: MS spectrum for dipropylene glycol at retention time of 19.20 min.


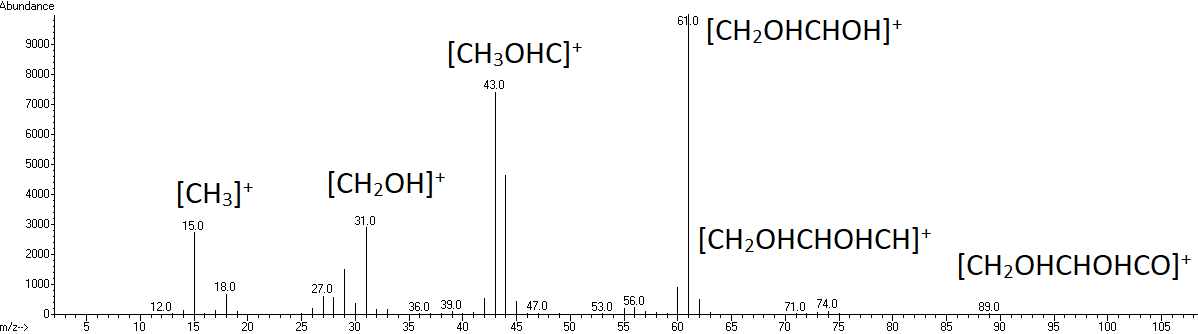

Supplementary Figure 11: MS spectrum for glycerol at retention time 22.10 min.


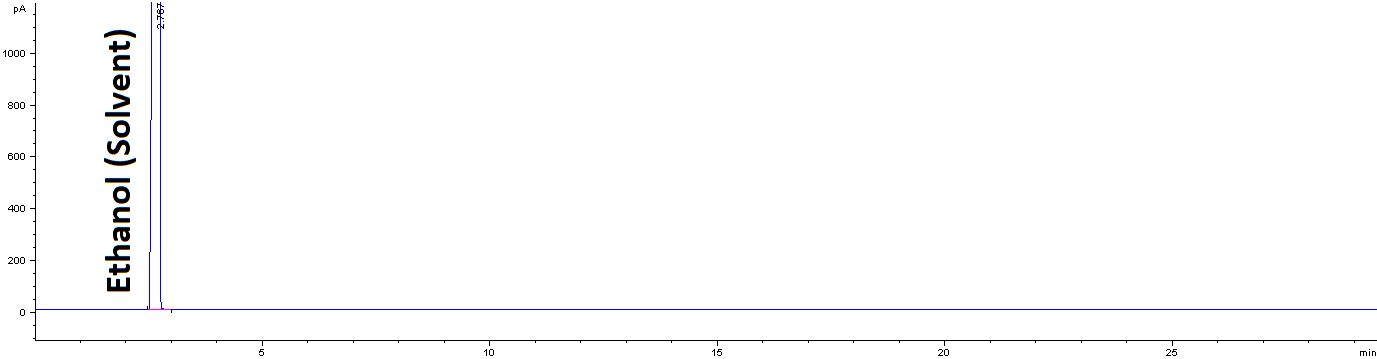


Supplementary Figure 12: Blank test with ethanol as solvent.

## Examples of calculation methods

The examples of the calculations after 8 hours conversion is shown in the following part. The yield of glycerol and liquid products was calculated in the C moles. The area peak of the targeted compound was divided by the peak of tetraethylene glycol (TEG) as the internal standard in order to avoid the inconsistent result from the GC-FID analyses.

1. **Example of glycerol conversion and kinetics rate constant calculation**

After the GC analysis was carried out, the obtained area peak of glycerol was divided by area peak of TEG. The value was then inserted in the equation of glycerol calibration curve (Supplementary Figure 12) to get the concentration of unconverted glycerol.

| $\frac{Area of glycerol}{Area of TEG}=\frac{137.2}{459}=0.298910675 (y-value)$ | (1) |
| --- | --- |
| $y= mx+c is equal to y= 0.2253x \left( Glycerol calibration curve \right)$ | (2) |
| Hence, $0.298910675=0.2253 x$  $x=1.326722927 \frac{mg}{mL} of glycerol in 10 mL of volumetric flask$ | (3) |
| Mass (diluted glycerol in 10 mL of volumetric flask)  *=* $1.326722927 \frac{mg}{mL} \times10 mL= 13.26722927 mg of glycerol$ | (4) |

Supplementary Figure 12: Glycerol calibration curve.

To get the mole of glycerol in actual concentration, the mass of diluted glycerol was divided by volume of actual concentration in micropipette and multiply with the volume of actual concentration of glycerol left after 8 hours of reaction.

| $Glycerol conc.\left( in 250 mL reactor \right)= Glycerol conc. \left( in 0.0001 L micropipette \right)$ | (5) |
| --- | --- |
| $\frac{Mass (mg)}{0.250 L}=\frac{13.26722927 mg}{0.0001 L}$ | (6) |
| $Therefore,$ |  |
| $Mole\left( glycerol \right)=\frac{\left( \frac{13.26722927mg}{0.0001 L}\times0.215 L \times\frac{{10}^{-3}g}{1 mg} \right)}{92.094\frac{g}{mol}}$ | (7) |
| $Mole\left( glycerol \right)=0.302529822 mole glycerol \times3 C mole$ | (8) |
| $Mole\left( glycerol \right)=0.907589467 in C mole$ | (9) |

Subsequently, glycerol conversion was calculated using the following equation.

| $Glycerol conversion \left( \% \right)$  $= \frac{Converted glycerol [Gly. in feed-Gly. in outlet \left( in C mole \right)]}{Total amount of glycerol in feed (in C mole)} \times100\%$  $= \frac{2.25 in C mole- 0.907589467 in C mole}{2.25 in C mole} \times100$  $= 59.66\% glycerol conversion$ | (10) |
| --- | --- |

For the kinetics rate constant, the graph of ln C_t_/C_i_ *versus* time was plotted based on the concentration of glycerol obtained from the GC-FID analysis. The slope of the graph gave the kinetics rate constant in the unit of h^-1^. The value was further divided by 3600 s for 8 hours to change the unit into s^-1^. The calculation is shown in (11).

| $Kinetics rate constant= \frac{0.0239}{h} \times\frac{1 h}{3600 s}$  $=$0.0664 × 10^-4^ *s*^-1^ | (11) |
| --- | --- |

1. **Example of product yield and selectivity calculation**

For instance, to attain the mole of 1,2-propanediol (in C mole), the similar steps of calculation from (1) to (9) were applied. The calibration curve from 1,2-propanediol with known concentration (Supplementary Figure 13) was used. The yield and selectivity of 1,2-propanediol were calculated using the following equations.

Supplementary Figure 13: 1,2-propanediol calibration curve.

| $Product yield \left( \% \right)$  $= \frac{Amount of product (in C mole)}{Total amount of glycerol in feed (in C mole)} \times100\%$  $= \frac{0.678083883 in C mole}{2.25 in C mole} \times100\%$  $= 30.14\% in C mole$ *of 1,2-propanediol* | (12) |
| --- | --- |

| $Product selectivity \left( \% \right)$  $= \frac{Amount of product (in C mole)}{Converted glycerol [Gly. in feed-Gly. in outlet \left( in C mole \right)]} \times100\%$  $= \frac{0.678083883 in C mole}{2.25 in C mole- 0.907589467 in C mole} \times100\%$  $= 50.51 \% in C mole$ | (13) |
| --- | --- |
